# Integrin-linked kinase modulates longevity and thermotolerance in *C. elegans* through neuronal control of HSF-1

**DOI:** 10.1111/acel.12189

**Published:** 2014-01-09

**Authors:** Caroline Kumsta, Tsui-Ting Ching, Mayuko Nishimura, Andrew E Davis, Sara Gelino, Hannah H Catan, Xiaokun Yu, Chu-Chiao Chu, Binnan Ong, Siler H Panowski, Nathan Baird, Rolf Bodmer, Ao-Lin Hsu, Malene Hansen

**Affiliations:** 1 Development, Aging and Regeneration Program, Sanford-Burnham Medical Research InstituteLa Jolla, CA, USA; 2 Department of Internal Medicine, Division of Geriatric and Palliative Medicine, University of Michigan Medical SchoolAnn Arbor, MI, USA; 3 The Glenn Center for Aging Research, The Salk Institute for Biological Studies, The Howard Hughes Medical InstituteLa Jolla, CA, USA; 4 Department of Molecular and Integrative Physiology, University of Michigan Medical SchoolAnn Arbor, MI, USA; * Institute of Biopharmaceutical Sciences, National Yang-Ming UniversityTaipei, Taiwan

**Keywords:** aging, *C. elegans*, heat-shock response, HSF-1, integrin signaling, PAT-4/ILK

## Abstract

Integrin-signaling complexes play important roles in cytoskeletal organization and cell adhesion in many species. Components of the integrin-signaling complex have been linked to aging in both *Caenorhabditis elegans* and *Drosophila melanogaster*, but the mechanism underlying this function is unknown. Here, we investigated the role of integrin-linked kinase (ILK), a key component of the integrin-signaling complex, in lifespan determination. We report that genetic reduction of ILK in both *C. elegans* and *Drosophila* increased resistance to heat stress, and led to lifespan extension in *C. elegans* without majorly affecting cytoskeletal integrity. In *C. elegans*, longevity and thermotolerance induced by ILK depletion was mediated by heat-shock factor-1 (HSF-1), a major transcriptional regulator of the heat-shock response (HSR). Reduction in ILK levels increased *hsf-1* transcription and activation, and led to enhanced expression of a subset of genes with roles in the HSR. Moreover, induction of HSR-related genes, longevity and thermotolerance caused by ILK reduction required the thermosensory neurons AFD and interneurons AIY, which are known to play a critical role in the canonical HSR. Notably, ILK was expressed in neighboring neurons, but not in AFD or AIY, implying that ILK reduction initiates cell nonautonomous signaling through thermosensory neurons to elicit a noncanonical HSR. Our results thus identify HSF-1 as a novel effector of the organismal response to reduced ILK levels and show that ILK inhibition regulates HSF-1 in a cell nonautonomous fashion to enhance stress resistance and lifespan in *C. elegans*.

## Introduction

Integrin-linked kinase (ILK) is a central intracellular component of the integrin-signaling complex, a multicomponent protein complex clustered at the plasma membrane. Integrin-mediated cell–matrix and cell–cell adhesion play critical roles in numerous biological processes, including cell migration, survival, and proliferation (Wu & Dedhar, 2001). Accordingly, dysregulation of ILK has been observed in many human pathologies, including cancers and cardiomyopathies (Knoll *et al*., 2007; McDonald *et al*., 2008). ILK is also essential for the early development of model organisms such as *C. elegans*, *Drosophila*, zebrafish, and mice (Williams & Waterston, 1994; Zervas *et al*., 2001; Postel *et al*., 2008; Lange *et al*., 2009).

Although complete deficiency of ILK confers lethality, analysis of animals with moderately reduced levels of ILK or other components of the integrin-signaling complex has revealed a novel function in aging. Specifically, genetic screens using RNA interference (RNAi) have shown that depletion of *pat-4*/ILK and its conserved binding partner *pat-6*/Parvin extends lifespan in *C. elegans* (Hansen *et al*., 2005). Similarly, a heterozygous mutation in β1-integrin, the binding partner of ILK, caused delayed behavioral aging and reduced mortality of *Drosophila* (Goddeeris *et al*., 2003). These observations suggest that integrin signaling may constitute a novel, conserved longevity pathway, yet the molecular underpinnings remain unknown.

Many characterized longevity mechanisms are known to confer increased resistance to cellular and environmental stressors, such as high temperature. Heat stress activates the conserved transcription factor heat-shock factor-1 (HSF-1) via a multistep process involving oligomerization, post-translational modification, and nuclear translocation. In the nucleus, HSF-1 upregulates the transcription of many genes encoding heat-shock proteins. Collectively, this universal systemic response to elevated temperatures is known as the heat-shock response (HSR). In *C. elegans,* the HSR involves a circuit of neurons, AFD and AIY, that sense temperature and communicate the systemic activation of HSF-1 (Prahlad *et al*., 2008). Notably, overexpression of HSF-1 is sufficient to extend the lifespan of *C. elegans* (Hsu *et al*., 2003), and overexpression of the HSF-1 target genes *hsp-16* and *Hsp70* promotes longevity in *C. elegans* and *Drosophila*, respectively (Tatar *et al*., 1997; Hsu *et al*., 2003; Walker & Lithgow, 2003). These findings underscore the pivotal role of HSF-1 and the HSR in organismal aging.

In this study, we investigated the relationship between ILK, the HSR, and longevity. We found that HSF-1 was required for the extended lifespan of *C. elegans* depleted of *pat-4*/ILK during adulthood. Moreover, inhibition of *pat-4*/ILK and several other components of the integrin-signaling complex in adult nematodes also increased their thermotolerance. We found that *pat-4*/ILK inhibition increased *hsf-1* transcription and translation, resulting in the induction of a subset of HSF-1 target genes. Activation of HSF-1 required the function of AFD and AIY thermosensory neurons, known to be essential for establishing the HSR, but PAT-4/ILK was not expressed in these neurons. Instead, we found PAT-4/ILK to be expressed in neighboring neurons, in addition to its known expression in body-wall muscle and gonad. Of these tissues, we found neurons, and possibly the gonad, but not muscle to be important for the beneficial effects of *pat-4*/ILK reduction. These findings are the first to demonstrate a direct link between ILK and HSF-1, and suggest that PAT-4/ILK systemically modulates stress resistance and longevity in a cell nonautonomous manner via thermosensory neurons.

## Results

### Reduction of *pat*-*4*/ILK during adulthood increases lifespan without affecting muscle integrity

**Figure 1:**
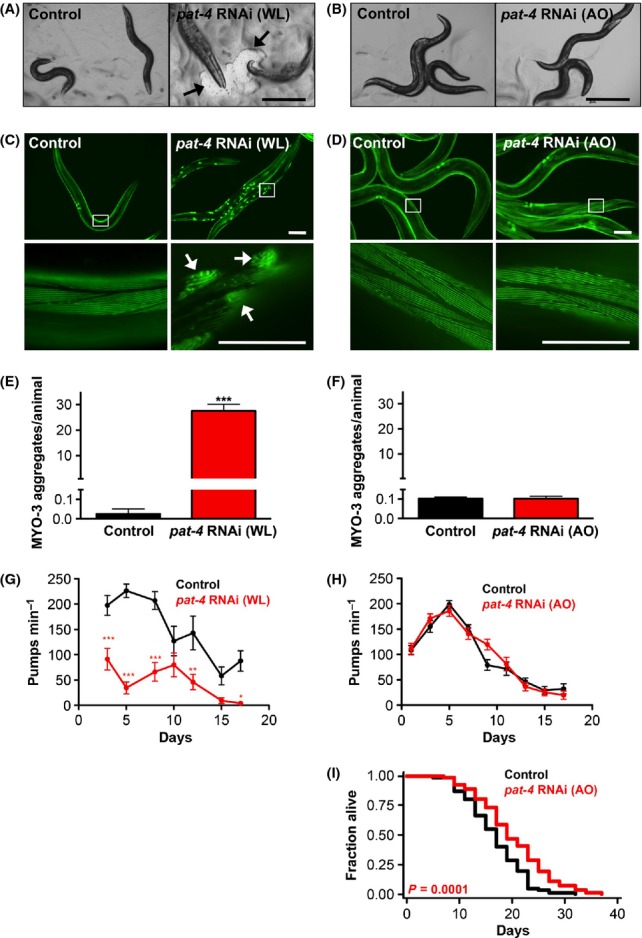
Inhibition of *pat*-*4*/ILK in adult *C. elegans* prolongs lifespan without inducing gross morphological defects. (A, B) Micrographs of N2 wild-type animals on day 2 (A) or day 5 (B) of adulthood. Animals were raised from hatching on bacteria containing empty vector or expressing *pat-4*/ILK dsRNA (whole-life, WL; A) or were raised on control bacteria and transferred to *pat-4*/ILK RNAi on day 1 of adulthood (adult-only, AO; B). Scale bars = 600 μm. Only WL *pat-4*/ILK RNAi caused paralysis, creating areas where the bacterial lawn was absent (A, arrows). (C, D) Fluorescence micrographs of animals expressing *myo-3p::gfp::myo-3*, taken on day 2 (C) or day 5 (D) of adulthood. Animals were raised as described for (A) and (B), respectively. Boxes indicate sections enlarged in the lower panels. Only WL *pat-4*/ILK RNAi resulted in MYO-3::GFP aggregates (C, arrows). Scale bars = 100 μm. (E, F) Quantification of MYO-3::GFP aggregates in animals raised as described for (C) and (D), respectively. Mean + SEM of N = 38–42 animals. ***P < 0.0001 (Student’s t-test). The experiment was repeated three times with similar results. (G, H) Pharyngeal pumping rates were measured in N2 wild-type animals raised as described for (A) and (B), respectively. Mean + SEM of N = 9–22. *P < 0.05, **P < 0.01, ***P < 0.001 of multiple comparison of RNAi treatment per day (Two-way anova: RNAi effect *F(1,110)* = 99.1, *P* < 0.0001, with an interaction of age and RNAi treatment *F(6,110)* = 3.61, *P =* 0.003). The experiment was repeated twice in N2 animals and twice in sterile CF512 animals with similar results. (I) Lifespan analysis of N2 animals transferred to bacteria expressing control (*gfp*) or *pat-4*/ILK dsRNA on day 1 of adulthood (AO). Similar lifespan extension was observed in a previous RNAi screen in which gene knockdown was initiated at the L4 larval stage (Curran & Ruvkun, 2007). Statistical analysis with log-rank (Mantel-Cox) test. The experiment was performed >10 times with similar results (see Table S1 for additional data). All experiments were performed at 20 °C.

We previously identified a role for *pat-4*/ILK in lifespan determination during a genome-wide RNAi screen in *C. elegans* to identify novel longevity genes (Hansen *et al*., 2005). This novel role for ILK in lifespan determination appears to be conserved, because *Drosophila* heterozygous *ilk* mutants are also long-lived (Nishimura *et al.,* 2014). To examine the novel longevity role of ILK in more detail, we reduced *pat-4/*ILK levels in *C. elegans* by feeding RNAi (*i.e*., using the *pat-4*/ILK bacterial RNAi clone previously identified in the screen mentioned above). PAT-4/ILK is essential for the development of *C. elegans* and plays an important role in muscle–cell integrity and cytoskeletal attachment in the embryo (Williams & Waterston, 1994; Mackinnon *et al*., 2002). Consistent with this, we found that animals fed *pat-4*/ILK dsRNA-expressing bacteria from hatching (*i.e*., whole-life RNAi treatment) showed adult-onset paralysis with minimal head movement (Fig. 1A), which was accompanied by irreversible aggregation and collapse of myosin filaments in the body-wall muscles (Fig. 1C,E) (Meissner *et al*., 2009). Because PAT-4/ILK is also expressed in the *C. elegans* pharynx (Mackinnon *et al*., 2002), we measured the pharyngeal pumping rate in animals subjected to *pat-4*/ILK RNAi from hatching. These animals showed markedly reduced pumping rates throughout their lifespan compared with animals fed bacteria containing empty vector (Fig. 1G). Such defects in pharyngeal pumping may reduce food intake and thus lead to dietary restriction, a longevity paradigm that extends lifespan in many species (Mair & Dillin, 2008). In principle, then, ‘mechanical’ dietary restriction could contribute to the longevity observed in *C. elegans* subjected to *pat-4*/ILK RNAi (Hansen *et al*., 2005; Table S1, Supporting information).

We next analyzed the function of PAT-4/ILK in adult nematodes by initiating *pat-4*/ILK RNAi on day 1 of adulthood (*i.e*., adult-only RNAi treatment); this treatment reduced *pat-4*/ILK mRNA levels by ~40% by day 3 (Fig. S1, Supporting information). This reduction in *pat-4*/ILK during adulthood increased the mean lifespan of *C. elegans* by ~10–30% (Fig. 1I; Table S1, Supporting information), similar to whole-life RNAi treatment (Hansen *et al*., 2005; Table S1, Supporting information). Notably, these animals did not become paralyzed or form myosin aggregates at any point during their lifespan, and their appearance was indistinguishable from that of control animals fed bacteria containing empty vector (Fig. 1B,D,F). This indicates that reducing PAT-4/ILK during adulthood extends lifespan without majorly affecting body-wall muscle integrity. Moreover, we found no difference in pharyngeal pumping rates between control animals and animals subjected to *pat-4*/ILK RNAi from day 1 of adulthood (Fig. 1H). This is in contrast with the pharyngeal pumping defect we observed in animals subjected to whole-life *pat-4*/ILK RNAi and implies that the longevity induced by *pat-4*/ILK reduction during adulthood is unlikely due to ‘mechanical’ dietary restriction. Thus, these data indicate that decreasing the expression of PAT-4/ILK during adulthood can extend the lifespan of *C. elegans* without affecting body-wall or pharyngeal muscle integrity.

### PAT-4/ILK reduction induces stress response genes

**Figure 2:**
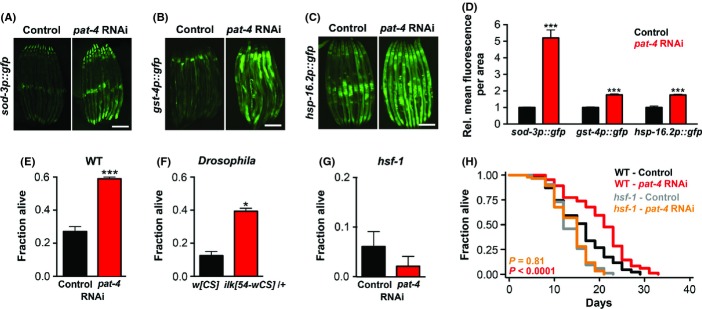
Inhibition of PAT-4/ILK in adult *C. elegans* leads to the induction of stress reporters, thermotolerance, and longevity via HSF-1. (A–C) Fluorescence micrographs (A–C) and quantification of relative GFP intensity (D) of wild-type animals expressing *sod-3p::gfp* (A), *gst-4p::gfp* (B), or *hsp-16.2p::gfp* (C). Animals were transferred to bacteria containing empty vector or expressing *pat-4*/ILK dsRNA on day 1, and images were captured on day 3. Exposure times were 1 s for *sod-3p::gfp* and *hsp-16.2p::gfp*, and 600 ms for *gst-4p::gfp*. Scale bars = 200 μm. For imaging of the *hsp-16.2p::gfp* reporter, animals were incubated for 1 h at 36 °C followed by 2 h at 20 °C. The experiments were repeated at least three times with similar results. N = 9–12. ***P < 0.001 (two-way anova). (E) Survival of N2 wild-type (WT) *C. elegans* after 8 h incubation at 36 °C. Animals were transferred to bacteria containing empty vector or expressing *pat-4*/ILK dsRNA on day 1, and survival was measured on day 3. Mean + SEM. **P < 0.01 (Student’s *t*-test). The experiment was performed a total of 13 times (see Table S2 for additional data). (F) Survival of 3-week-old *ilk*^*54-wCS*^/+ and *w*^*CS*^ (control) *Drosophila* was measured after 85 min incubation at 36 °C. Mean + SEM of N = 40 *P < 0.05 (Student’s t-test). The experiment was repeated three times with similar results. (G) Survival of *hsf-1(sy441)* mutants after 7 h incubation at 36 °C. Animals were transferred to bacteria containing empty vector or expressing *pat-4*/ILK dsRNA on day 1, and survival was measured on day 3. Mean + SEM. *P < 0.05, **P < 0.01 (Student’s *t*-test). See Table S4 for additional data. (H) Lifespan analysis of WT animals and *hsf-1(sy441)* mutants transferred to bacteria expressing control (*gfp*) or *pat-4*/ILK dsRNA on day 1 of adulthood. Statistical analysis with log-rank (Mantel-Cox) test. The experiment was repeated three times with similar results (see Table S1 for additional data). All experiments were performed at 20 °C.

**Figure 3:**
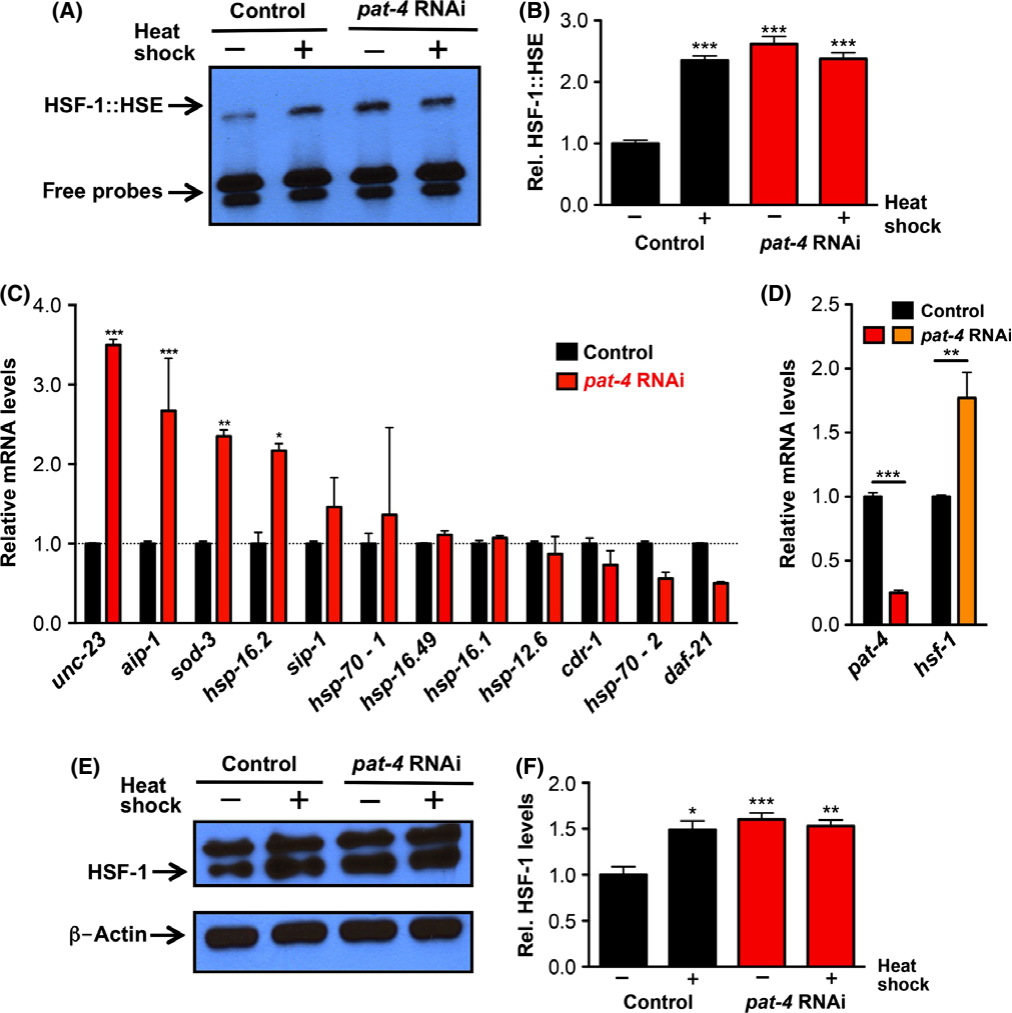
HSF-1 levels and activity are increased in *C. elegans* in response to *pat-4*/ILK reduction. (A) Representative electrophoretic mobility shift assay performed on nuclear extracts from 2-day-old N2 wild-type animals raised from hatching on bacteria containing empty vector or expressing *pat-4*/ILK dsRNA. Animals were either unstressed or exposed to heat shock at 37 °C for 90 min before harvest. Arrows indicate HSF-1 bound to biotin-labeled oligonucleotides containing the heat-shock element (HSF-1::HSE) or unbound oligonucleotides (free probes). The experiment was performed three times with similar results. (B) Quantification of electrophoretic mobility shift assays. Results are the Mean + SD of three independent experiments (including that shown in (A), normalized to the control. ***P < 0.001 (two-way anova). (C–D) Quantitative RT–PCR of total RNA from 2-day-old N2 animals raised from hatching on bacteria containing empty vector or expressing *pat-4*/ILK dsRNA. mRNA levels of known HSF-1 target genes (C) or *pat-4*/ILK and *hsf-1* (D) were normalized to the housekeeping genes *ama-1* and *nhr-23*. We confirmed that *pat-4*/ILK RNAi did not affect expression of an *hsp-70p(C12C8.1)::gfp* construct (data not shown), corroborating these results. Mean + SEM of three independent experiments. *P < 0.05, **P < 0.01, ***P < 0.001 (one-way anova). (E) Western blot analysis of HSF-1 in total protein extracts from 2-day-old N2 animals raised from hatching on bacteria containing empty vector or expressing *pat-4*/ILK dsRNA. Animals were either unstressed or exposed to heat shock at 37 °C for 90 min before harvest. The experiment was performed five times. (F) Quantification of relative HSF-1 protein levels. Results are the Mean + SD of five independent experiments (including that shown in E), normalized to the loading control (β-actin), and compared to the unstressed animals expressing empty vector. *P < 0.05, **P < 0.01, ***P < 0.001 (two-way anova).

Many organisms, including *C. elegans,* exhibit increased resistance to stress when subjected to conserved lifespan-extending treatments or pertubations (Johnson *et al*., 2001). To determine whether inhibition of *pat-4*/ILK might evoke a stress response, we examined the induction of three commonly used stress reporters in *C. elegans*: the oxidative stress reporters *sod-3p::gfp* (Libina *et al*., 2003) and *gst-4p::gfp* (Link & Johnson, 2002), and heat-stress reporter *hsp-16.2p::gfp* (Mendenhall *et al*., 2012). We found that expression of all three reporters was increased in multiple tissues, most notably in the intestine, in 3-day-old animals subjected to *pat-4*/ILK RNAi from day 1 of adulthood (Fig. 2A–D). Consistently, we found the mRNA levels of *sod-3* and *hsp-16.2* significantly upregulated upon reduction in *pat-4*/ILK (Fig. 3C). Thus, inhibition of *pat-4*/ILK induced expression of genes typically upregulated in response to environmental stressors, consistent with the mobilization of a stress response.

### Inhibition of *pat*-*4*/ILK confers thermotolerance in both *C. elegans* and *Drosophila*

We next asked whether inhibition of *pat-4*/ILK could induce stress resistance in *C. elegans* by examining the response to increased temperature. We focused on this type of stress as heat stress causes disruption of the cytoskeleton in several species (Walter *et al*., 1990; Fisher *et al*., 1996), and PAT-4/ILK is localized at this site in *C. elegans* (Mackinnon *et al*., 2002). To test survival at elevated temperatures, we subjected animals to *pat-4*/ILK RNAi from day 1 of adulthood and on day 3 incubated them at 36 °C for 8 h. Survival was scored by the response to a gentle stimulus to the head region. We found that animals subjected to *pat-4*/ILK RNAi prior to the heat shock were indeed more resistant to elevated temperatures than animals fed control bacteria (Fig. 2E, Table S2, Supporting information).

Because ILK depletion also extends the lifespan of *Drosophila* (Nishimura *et al.,* 2014), we next asked whether the thermotolerance phenotype might represent a conserved response to ILK inhibition. For this, vials containing 3-week-old *Drosophila ilk* outcrossed heterozygotes or wild-type flies were submerged in a 36 °C water bath for 85 min. Under these conditions, we observed that both male and female flies expressing reduced levels of *ilk* were significantly more thermotolerant than their wild-type counterparts (Fig. 2F), consistent with our findings in *C. elegans*. These observations indicate that the elevated thermotolerance exhibited by animals with reduced ILK levels may be a conserved protective mechanism and could contribute to the extended lifespan observed in both *C. elegans* (Hansen *et al*., 2005; Curran & Ruvkun, 2007) and *Drosophila* (Nishimura *et al*., 2014).

### Stress resistance is influenced by many integrin-signaling complex components

The integrin-signaling complex acts as a physical link between the extracellular matrix and the cytoskeleton. To identify other integrin-signaling complex components that might affect thermotolerance in *C. elegans*, we used RNAi to systematically reduce the levels of such integrin-complex components in adult animals and then analyzed the effects on thermotolerance. We also tested the effects of RNAi-depleting other cytoskeletal components that, similar to whole-life *pat-4*/ILK RNAi treatment (Fig. 1A,C), can induce detachments in the cytoskeletal lattice, as visualized by MYO-3 aggregate accumulation (Meissner *et al*., 2009). In repeated experiments, we identified several RNAi clones that consistently induced thermotolerance, including *pat-6*/Parvin, *deb-1*/Vinculin, and *unc-89*/Obscurin (Table S2, Supporting information). We note that a *pat-6*/Parvin RNAi clone was identified in the previously mentioned RNAi-longevity screen (Hansen *et al*., 2005), and we found that *pat-6*/Parvin RNAi treatment in adult animals was capable of extending *C. elegans* lifespan to a similar extent as *pat-4*/ILK RNAi (data not shown). Other RNAi clones tested also induced thermotolerance but with more variable results; for example, *kin-32*/FAK (Focal Adhesion Kinase), *unc-98*, *unc-95*/paxillin, *unc-52*/perlecan, *myo-3*/myosin heavy chain, and *unc-97*/PINCH (Tables S2 and S3, Supporting information). Taken together, our data suggest that thermotolerance can be increased by reducing the expression of a number of integrin-complex components, in addition to *pat-4*/ILK. Interestingly, most of the RNAi clones we tested caused some degree of disruption of PAT-4/ILK expression (*i.e.,* in M-lines and dense bodies in body-wall muscle cells) when fed to animals from hatching (Fig. S2, Table S3, Supporting information), suggesting that their thermotolerant phenotypes may be mediated, at least in part, via *pat-4*/ILK.

### HSF-1 is required for thermotolerance and longevity induced by *pat-4*/ILK reduction

We next sought to determine how PAT-4/ILK may regulate thermotolerance in *C. elegans*. We focused on analyzing the role of three major stress-regulating transcription factors, DAF-16/FoxO, SKN-1/Nrf, and HSF-1, which regulate the genes we assayed earlier, that is, *sod-3*, *gst-4*, and *hsp-16.2,* respectively (see also Fig. 2A–C). For this, we examined the expression of the genes in the transcription factor mutants *daf-16(mu86), skn-1(RNAi),* and *hsf-1(sy441*). *pat-4*/ILK RNAi failed to increase in the expression of *sod-3p::gfp*, *gst-4p::gfp*, and *hsp-16.2p::gfp* in these mutants (Fig. S3B,D Supporting information), suggesting that depletion of *pat-4*/ILK may induce expression of heat- and oxidative stress response genes through the regulation of DAF-16/FoxO, SKN-1/Nrf, and HSF-1. To determine whether these transcription factors were required for the thermotolerant phenotype induced by *pat-4*/ILK RNAi, we measured the survival of *daf-16*/FoxO, *skn-1*/Nrf, and *hsf-1* mutants exposed to elevated temperatures. Interestingly, *pat-4*/ILK RNAi during adulthood induced thermotolerance in *daf-16(mu86)* mutants and *skn-1(zu135)* mutants (Table S4, Supporting information), but had no effect on thermotolerance in *hsf-1(sy441)* mutants (Fig. 2G, Table S4, Supporting information), even though *pat-4*/ILK levels can be potently reduced in *hsf-1* mutants (Fig. S4, Supporting information). We also eliminated the possibility that *hsf-1* mutants were inherently incapable of elevated thermotolerance by showing that *hsf-1(sy441)* mutants subjected to RNAi targeting the insulin/IGF-1 receptor *daf-2*/InR indeed displayed increased resistance to heat stress (Table S4, Supporting information).

Although *daf-16/*FoxO and *skn-1*/Nrf1 were not required for thermotolerance caused by *pat-4*/ILK inhibition, we noted that both the DAF-16/FoxO target gene *sod-3* and the SKN-1/Nrf1 target gene *gst-4* were induced by *pat-4*/ILK RNAi. We therefore tested whether the *sod-3p::gfp* and *gst-4p::gfp* reporters could be induced in *hsf-1(sy441)* mutants upon inhibition of *pat-4*/ILK and found that *hsf-1* was at least partially required for the induction of both *sod-3p::gfp* and *gst-4p::gfp* (Fig. S3C,D, Supporting information). These results demonstrated that *sod-3* and *gst-4* are regulated not only by DAF-16/FoxO and SKN-1/Nrf, but also by HSF-1, at least in response to *pat-4*/ILK reduction. We note that *hsp-16.2* can also be regulated by DAF-16/FoxO to some extent (Hsu *et al*., 2003). In contrast to the stress-reporter induction, we found that *daf-16*/FoxO and *skn-1*/Nrf were not required for *pat-4*/ILK RNAi-induced thermotolerance (Table S4, Supporting information). Additionally, the lifespan extension of animals subjected to whole-life or adult-only *pat-4*/ILK RNAi is *daf-16*/FoxO-independent (Hansen *et al*., 2005; Curran & Ruvkun, 2007). It is possible that additional stress responses, besides thermotolerance are engaged in *pat-4*/ILK RNAi-treated animals via DAF-16/FoxO or other transcription factors. Collectively, these data demonstrate that *hsf-1* is required for the induction of stress-responsive genes and thermotolerance in animals with reduced PAT-4/ILK levels.

To determine whether *hsf-1* was similarly required for the longevity of animals subjected to *pat-4*/ILK RNAi, we performed epistasis experiments with *hsf-1(sy441)* mutant animals. Reduction in *pat-4*/ILK during adulthood did not extend the lifespan of *hsf-1(sy441)* mutants, whereas inhibition of *daf-2*/InR did (Fig. 2H, Table S1, Supporting information). The latter result further links stress responses to longevity and supports the possibility that *hsf-1-*dependent induction of stress-response genes could underlie the novel lifespan-determining function of PAT-4/ILK, at least in *C. elegans*.

### PAT-4/ILK reduction increases HSF-1 activity

Having shown that HSF-1 was required for the extended longevity and thermotolerance of animals with reduced PAT-4/ILK levels, we next asked whether PAT-4/ILK could regulate HSF-1 activity. For this, we performed electrophoretic mobility shift assays to measure the binding of HSF-1 to a sequence encompassing the heat-shock element (HSE) of a target gene (Voellmy, 2004; Chiang *et al*., 2012). We found that HSF-1–HSE binding was not only increased in nuclear extracts from *C. elegans* raised on empty-vector bacteria and exposed to heat shock of 37 °C for 90 min, as previously reported (Hsu *et al*., 2003; Chiang *et al*., 2012), but also in nuclear extracts from *C. elegans* raised under normal temperature conditions (20 °C) on bacteria expressing *pat-4*/ILK dsRNA (Fig. 3A,B). There was no further increase in the binding of HSF-1 upon additional heat shock (Fig. 3A,B), which indicated that the *pat-4*/ILK RNAi treatment had saturated the binding of HSF-1 to the available HSE elements or that a similar process of HSF-1 activation occurs in animals upon heat shock and reduction in *pat-4*/ILK.

To verify that increased binding of HSF-1 to HSEs translated into increased transcriptional activity, we performed quantitative RT–PCR of HSF-1 target genes known to be regulated in response to heat shock from animals raised at 20 °C and subjected to *pat-4*/ILK RNAi. We found that *pat-4*/ILK inhibition upregulated the HSF-1 target genes *unc-23* and *aip-1* (Fig. 3C) (GuhaThakurta *et al*., 2002; Hsu *et al*., 2003) as well as *sod-3* and *hsp-16.2*, corroborating our results with the stress reporter genes (Figs 3C, and 2A,C). In contrast, other HSF-1-regulated genes, such as *sip-1* and *hsp-70* (C12C8.1 and F44E5.4), and several small heat-shock proteins (*hsp-16.1, hsp-16.49,* and *hsp-12.6*) were unchanged, suggesting that only a subset of heat shock-inducible HSF-1 target genes are relevant for the increased longevity and thermotolerance observed in *C. elegans* raised at normal temperature with reduced *pat-4*/ILK levels (Fig. 3C).

We next investigated the mechanism by which *pat-4*/ILK RNAi upregulated HSF-1 target genes by analyzing *hsf-1* gene expression. Interestingly, *hsf-1* mRNA levels were significantly increased by *pat-4*/ILK RNAi (Fig. 3D). This was unexpected because we had previously found that neither heat-shock treatment nor reduction in *daf-2*/InR in *C. elegans* increase *hsf-1* transcription (Hsu *et al*., 2003; Chiang *et al*., 2012). Consistent with the increase in *hsf-1* transcription, we found that animals subjected to *pat-4*/ILK RNAi contained increased levels of HSF-1 protein, and the level was comparable with that observed in control animals following heat shock (Fig. 3E,F). HSF-1 protein expression in animals subjected to *pat-4*/ILK RNAi was not further increased by heat shock (Fig. 3E,F). Thus, reduction in *pat-4*/ILK induced transcription and translation of *hsf-1*, and upregulated the expression of a subset of known HSF-1 target genes in *C. elegans* without any temperature insults. Taken together, these observations suggest that a specialized HSF-1-mediated heat-stress response is activated in animals with reduced levels of *pat-4*/ILK.

### Thermosensory neurons are required for *pat-4*/ILK-regulated thermotolerance and longevity

**Figure 4:**
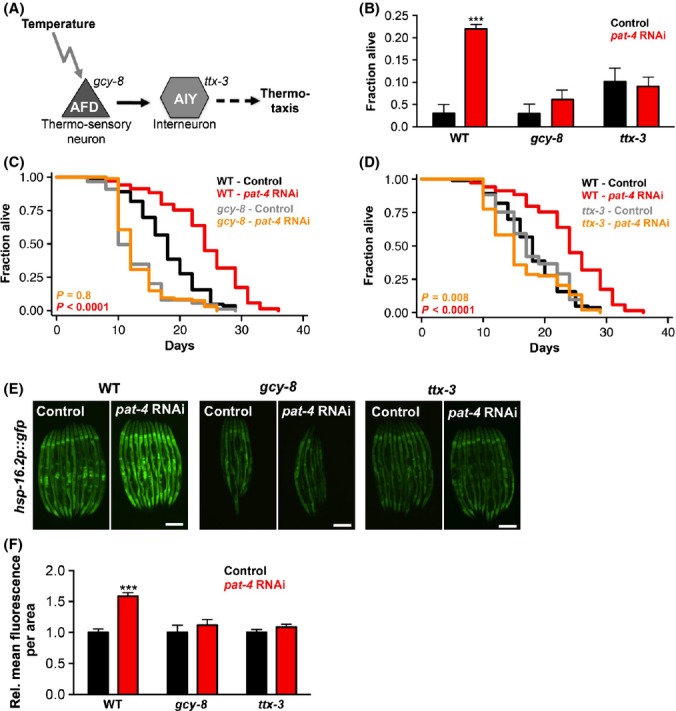
Inhibition of *pat-4*/ILK in adult *C. elegans* requires functional thermosensory neurons to promote thermotolerance, longevity, and induction of the stress reporter *hsp-16.2p::gfp*. (A) Schematic model of temperature sensing. The AFD neurons sense temperature via the nucleotide-gated channel TAX-2/TAX-4 (not shown), which engages the guanylyl cyclase GCY-8. This event activates the LIM homeodomain protein TTX-3 in the interneurons AIY to ultimately direct thermotactic movement (Mori & Ohshima, 1995). (B) Survival of N2 wild-type (WT) animals, and *gcy-8(oy44)* and *ttx-3(ks5)* mutants after 8 h incubation at 36 °C. Animals were transferred to bacteria containing empty vector or expressing *pat-4*/ILK dsRNA on day 1 and survival was measured on day 3. Mean + SEM. ***P < 0.001 (two-way anova). The experiment was repeated three times with similar results (see Table S5 for additional data). (C–D) Lifespan analysis of WT animals, *gcy-8(oy44)* mutants (C), and *ttx-3(ks5)* mutants (D) transferred to bacteria expressing control (*gfp*) or *pat-4*/ILK dsRNA on day 1 of adulthood. The same wild-type control is presented in (C) and (D) because the three strains were tested in the same experiment. Statistical analysis with log-rank (Mantel-Cox) test. The experiment was repeated three times with similar results (see Table S1 for additional data). All experiments were carried out at 20 °C. (E) Fluorescence micrographs of wild-type animals, *gcy-8(oy44)* mutants, and *ttx-3(ks5)* mutants expressing *hsp-16.2p::gfp*. Animals were transferred to bacteria containing empty vector or bacteria expressing *pat-4*/ILK dsRNA on day 1, and images were captured on day 3. Exposure time was 1 s. Prior to imaging, animals were incubated for 1 h at 36 °C followed by 2 h at 20 °C. Scale bars = 200 μm. (F) Quantification of relative GFP intensity of the animals shown in (E). N = 7–12. ***P < 0.001 (two-way anova). The experiment was repeated three times with similar results.

Upon exposure to high temperatures, *C. elegans* elicit a canonical heat-shock response (HSR) that is regulated systemically by signals from a neuronal circuit that senses temperature and regulates thermotaxis (Fig. 4A) (Jeong *et al*., 2012). Specifically, the AFD thermosensory neurons (*i.e*., the neuronal pair AFDR and AFDL) detect temperature through a nucleotide-gated channel TAX-2/TAX-4, which requires the upstream function of several guanylyl cyclases, one of which is GCY-8. The sensory input is signaled through the AIY interneurons, (*i.e*., the neuronal pair AIYR and AIYL) which require the LIM homeodomain protein TTX-3 for function (Mori & Ohshima, 1995). Upon perception of heat shock, the AFD and AIY neurons regulate a HSR in distal tissues of the animals (Prahlad *et al*., 2008; Prahlad & Morimoto, 2011). To determine whether the AFD and AIY neurons play a similar role in *pat-4*/ILK-modulated thermotolerance, we examined the loss-of-function mutants *gcy-8* and *ttx-3* after feeding with *pat-4*/ILK dsRNA-expressing or control bacteria. We found that *pat-4*/ILK RNAi treatment during adulthood failed to increase thermotolerance (Fig. 4B, Table S5, Supporting information) as well as longevity (Fig. 4C,D, Table S1, Supporting information) of the *gcy-8(oy44)* and *ttx-3(ks5)* mutants compared with wild-type animals. These observations confirm that, as with the canonical HSR, functional thermosensory neuron AFD and interneuron AIY are required for thermotolerance and longevity in animals with reduced *pat-4*/ILK levels.

To further explore the role of the AFD thermosensory neurons and AIY interneurons, we introduced the *pat-4*/ILK-responsive stress reporter *hsp-16.2p::gfp* into the *gcy-8(oy44)* and *ttx-3(ks5)* mutants. In contrast to wild-type animals, *gcy-8(oy44)* and *ttx-3(ks5)* mutants failed to induce *hsp-16.2::gfp* expression, including in the intestine (Fig. 4E–F). Consistent with this observation, transcription of *hsp-16.2* and of other tested HSF-1 target genes was unchanged in *gcy-8(oy44)* and *ttx-3(ks5)* mutants subjected to *pat-4*/ILK RNAi (Fig. S5, Supporting information). Moreover, *hsf-1* transcript levels were not increased in these mutants (Fig. S5, Supporting information). Thus, functional AFD and AIY neurons are likely required for the effects of *pat-4*/ILK reduction not only for the expression of *hsf-1* but also for the induction of *hsf-1* target genes in the intestine, a distal tissue not reported to express PAT-4/ILK. Taken together with our thermotolerance- and lifespan analyses on *gcy-8* and *ttx-3* mutants, these results indicate that *pat-4*/ILK mediates effects via the AFD thermosensory neurons and the AIY interneurons.

### *pat-4*/ILK functions in neurons, but not in muscle, to mediate longevity

**Figure 5:**
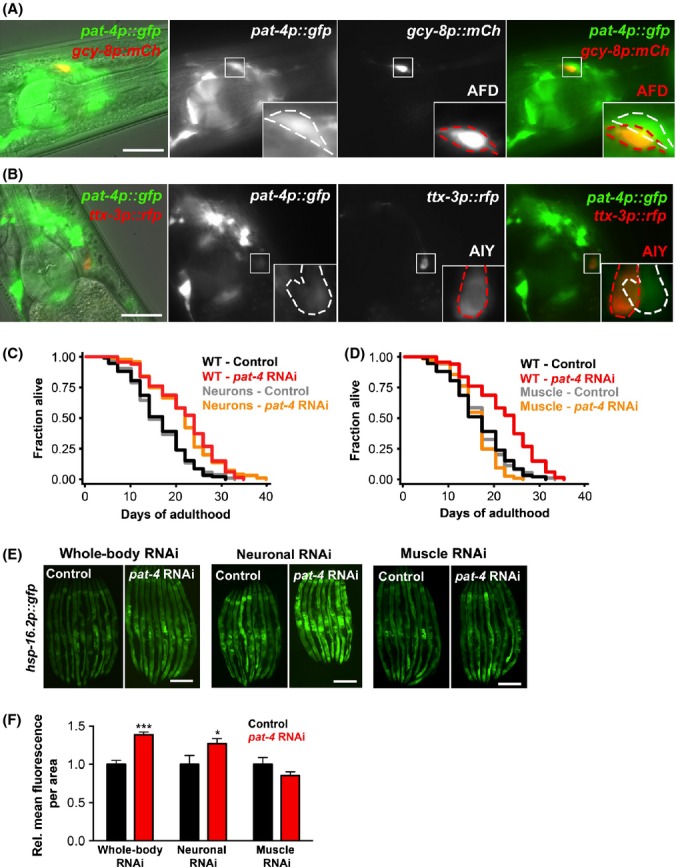
Inhibition of *pat-4*/ILK in neurons, but not in muscle, of adult *C. elegans* promotes longevity and induction of the stress reporter *hsp-16.2p::gfp*. (A–B) Micrographs showing the pharynx of animals co-expressing *pat-4p::gfp* and *gcy-8p::mCherry* (A) or *ttx-3p::rfp* (B) on day 1 of adulthood. Images of an overlay of the fluorescence channels with DIC (first column), of the fluorescence channels (second and third columns), and an overlay of the fluorescence channels (fourth column) are shown. The outline of the single cells expressing *pat-4p::gfp* (white) and the cell body of one of the AFD (A) or AIY (B) neurons (red) are highlighted in the magnified insets. Scale bars = 20 μm. (C–D) Lifespan analysis of N2 wild-type (WT) animals, neuronal-specific RNAi strain (*sid-1; rab-3p::sid-1*) (C), and muscle-specific RNAi strain (*sid-1; myo-3p::sid-1*) transferred to bacteria expressing empty vector or *pat-4*/ILK dsRNA on day 1 of adulthood. The same wild-type control is presented in (C) and (D) because the three strains were tested in the same experiment. Statistical analysis with log-rank (Mantel-Cox) test. The experiment was repeated three times with similar results (see Table S1 for additional data and Fig. S5 for information on RNAi effects in the *sid-1* transgenic strains). All experiments were carried out at 20 °C. (E) Fluorescence micrographs of wild-type animals (‘whole-body’ RNAi), neuronal-specific RNAi strain (*sid-1; rab-3p::sid-1*) and muscle-specific RNAi strain (*sid-1; myo-3p::sid-1*) expressing *hsp-16.2p::gfp*. Animals were transferred to bacteria containing empty vector or bacteria expressing *pat-4*/ILK dsRNA on day 1 and images were captured on day 3. Exposure time was 1 s. Prior to imaging, animals were incubated for 1 h at 36 °C followed by 2 h at 20 °C. Scale bars = 200 μm. (F) Quantification of relative GFP intensity of the animals shown in (E). N = 9–14. *P < 0.05, ***P < 0.001 (two-way anova). The experiment was repeated three times with similar results.

To learn more about how PAT-4/ILK elicits organismal effects on lifespan and thermotolerance via the AFD thermosensory neurons and AIY interneurons, we next investigated where in the animal PAT-4/ILK functions. As a start, we asked whether *pat-4*/ILK acted cell autonomously in these thermosensory neurons. We examined the expression of *pat-4*/ILK specifically in AFD and AIY neurons using a transcriptional *pat-4p::gfp* reporter construct (Dupuy *et al*., 2007) expressed in combination with *gcy-8p::mCherry* or *ttx-3p::rfp* constructs. Surprisingly, we found that *pat-4p::gfp* was neither expressed in AFD (Fig. 5A) nor in AIY neurons (Fig. 5B), but was present in adjacent cells, which may be ADF and/or AIM neurons (chemosensory/oxygensensory neurons and interneurons, respectively). Importantly, we detected no difference in the thermotactic behavior of control animals and those subjected to *pat-4*/ILK RNAi in adulthood (Fig. S6, Supporting information). Thus, although the stress response mediated by *pat-4*/ILK reduction requires the presence of functional AFD and AIY neurons (Fig. 4E–F), these thermosensory neurons may not directly mediate the effects on thermotolerance and longevity, suggesting that PAT-4/ILK functions cell-nonautonomously outside the AFD and AIY neurons.

**Figure 6:**
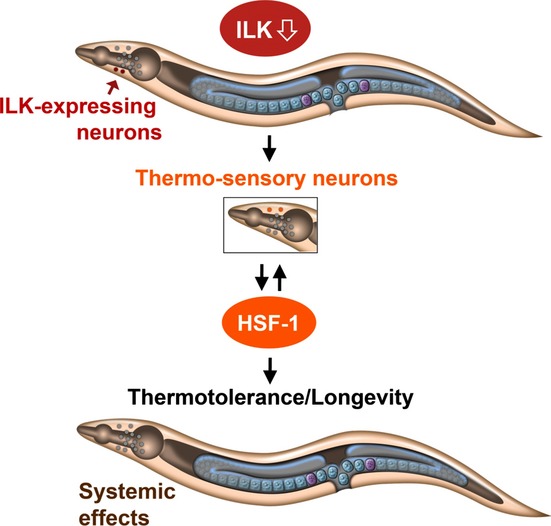
Model for how PAT-4/ILK modulates thermotolerance and longevity in *C. elegans*. In this organism, depletion of *pat-4*/ILK in PAT-4/ILK-expressing neurons, localized for example, in the head region of the animal as shown here*,* activates the transcription factor HSF-1 via specific thermosensory neurons also localized in the head (which do not express *pat-4*/ILK), thereby ensuring the induction of an HSF-1 meditated stress response in distal tissues (*e.g.,* the intestine), as well as thermotolerance and longevity. It is possible that additional PAT-4/ILK-expressing tissues/organs, such as the somatic gonad, are also important for the longevity function of PAT-4/ILK in *C. elegans*. A notable exception may be the body-wall muscle, as reduction in *pat-4*/ILK in this tissue failed to induce longevity and stress responses; see text for details.

PAT-4/ILK is expressed in several mechanosensory neurons different from the cells we identified above, as well as in body-wall muscle cells, the pharynx and in somatic gonad structures (Mackinnon *et al*., 2002). To specifically investigate the role of PAT-4/ILK in neurons and muscle, we used tissue-specific *sid-1* RNAi strains. *sid-1* mutants are deficient in transporting dsRNA molecules from one tissue to another (Winston *et al*., 2002) but have been engineered to express the SID-1 dsRNA transporter under a neuronal- (*sid-1(qt9); rab-3p::sid-1*) or body-wall-muscle-specific promoter (*sid-1(qt9); myo-3p::sid-1*) (see legend for Fig. S5, Supporting information for more information on RNAi effects in these animals). Using these strains, we found that adult-only *pat-4*/ILK RNAi treatment significantly increased the lifespan of animals when *pat-4*/ILK was reduced in neurons, indicating that PAT-4/ILK-expressing neurons are important in conferring the lifespan extension (Fig 5C, Table S1, Supporting information). Consistent with these observations, we found that *pat-4*/ILK RNAi in neurons induced *hsp-16.2p::gfp* expression, including in the intestine (Fig. 5E–F). In contrast, reduction in *pat-4*/ILK in body-wall muscles neither increased lifespan (Fig. 5D, Table S1, Supporting information) nor induced the *hsp-16.2p::gfp* reporter (Fig. 5E–F), even though *pat-4*/ILK levels were potently reduced in the muscle-specific RNAi strain in response to this RNAi treatment (Fig. S5C, Supporting information). In summary, our analyses using tissue-specific RNAi strains indicate that neurons, but not muscle, constitute a critical organ in which *pat-4*/ILK functions to cause systemic effects on the animal. As the AFD thermosensory neurons and the AIY interneurons are similarly required for these effects but do not express PAT-4/ILK, our results indicate that *pat-4*/ILK reduction causes thermotolerance and lifespan extension as well as stress responses in tissues not expressing PAT-4/ILK (such as the intestine) via cell nonautonomous signaling (see model in Fig. 6).

## Discussion

Inhibition of components of the integrin-signaling complex extends lifespan in both *Drosophila* and *C. elegans* (Goddeeris *et al*., 2003; Hansen *et al*., 2005; Curran & Ruvkun, 2007; Nishimura *et al*., 2014) raising the possibility that integrin signaling constitutes a conserved longevity pathway. In this study, we characterized the molecular mechanisms by which one component of this complex, PAT-4/ILK, modulates lifespan in *C. elegans*. Reduced ILK levels rendered both *C. elegans* and *Drosophila* more resistant to heat stress, suggesting that increased thermotolerance may be one mechanism by which ILK modulates aging. We also identified HSF-1, the key regulator of the canonical HSR, as a critical effector of the thermotolerance induced by *pat-4*/ILK depletion, providing the first connection between HSF-1 and ILK. Specifically, we found that ILK regulates the expression of *hsf-1* and of specific stress response genes via a neuronal circuit that includes neurons involved in thermosensation to establish a systemic stress response that may mediate, or accompany, *pat-4*/ILK RNAi-induced lifespan extension in *C. elegans* (Fig. 6).

The observation that a reduction in PAT-4/ILK levels increased *hsf-1* transcription in *C. elegans* was particularly noteworthy, as neither heat stress nor reduced insulin/IGF-1 signaling increases *hsf-1* mRNA levels (Hsu *et al*., 2003; Chiang *et al*., 2012), and increased *hsf-1* transcript levels have so far only been demonstrated in breast cancer tumor cells (Santagata *et al*., 2011). To our knowledge, our study is the first to show that transcriptional regulation of *hsf-1* is functionally relevant to an organismal stress response.

The HSF-1-mediated stress response in *C. elegans* requires the thermosensory neurons AFD and AIY for the systemic induction of multiple HSF-1 target genes, such as heat-shock proteins necessary for organismal proteostasis (Prahlad *et al*., 2008; Prahlad & Morimoto, 2011). We found that lifespan extension, thermotolerance, HSF-1 upregulation, and *hsp-16.2* induction following *pat-4*/ILK inhibition were all dependent on the function of the AFD and AIY neurons, similar to events occurring during a canonical heat-shock response (HSR). However, *pat-4*/ILK RNAi upregulated only a subset of known heat-inducible HSF-1 targets and induced transcription of *hsf-1* itself in animals raised at normal temperatures, implying that the loss of *pat-4*/ILK induced a noncanonical HSR. Interestingly, differential activation of HSF-1 targets in response to heat stress has been demonstrated in certain cell types; for example, human retinoblastoma cells fail to upregulate Hsp70 upon heat shock, even though HSF-1 is activated and Hsp90 is induced (Mathur *et al*., 1994). Thus, atypical or specialized HSRs can be mounted at least in some mammalian cells, similar to our demonstration of a *pat-4*/ILK RNAi-induced HSR in *C. elegans*.

While PAT-4/ILK was not expressed in the AFD and AIY thermosensory neurons, we observed that depletion of *pat-4*/ILK from other neurons was sufficient to increase *C. elegans* lifespan and induce the expression of the HSF-1-target gene *hsp-16.2*. In contrast, the main site of PAT-4/ILK expression, the body-wall muscles, did not seem to be important for the beneficial organismal effects of PAT-4/ILK. PAT-4/ILK-expressing neurons may therefore be a major tissue from which PAT-4/ILK reduction conveys organismal effects on thermotolerance and longevity. We note that it is possible that additional PAT-4/ILK-expressing tissues may contribute as well, as we found that gonad-less *gon-2*(q3888) mutants, which lack all gonad structures when raised at the nonpermissive temperature (*i.e*., somatic gonad and germline), failed to become thermotolerant upon reduction in *pat-4*/ILK (Table S6, Supporting information). Future experiments are needed to fully clarify how PAT-4/ILK-expressing tissues contribute to *C. elegans* lifespan determination. Likewise, it will be interesting to elucidate the signals that are required for the intertissue communication between PAT-4/ILK-expressing neurons (and possibly other sites), and AFD and AIY thermosensory neurons, that ultimately regulate the systemic HSF-1-mediated stress response observed in *C. elegans* with reduced PAT-4/ILK levels.

How could reduced levels of PAT-4/ILK increase thermotolerance and extend organismal lifespan in *C. elegans* in an *hsf-1-*dependent fashion? One possible mechanism could include alterations in the signaling pathways regulated by ILK downstream of the integrin-signaling complex (Qin & Wu, 2012). For example, HSF-1 might be negatively regulated by the ILK substrate glycogen synthase 3 (GSK3), as observed, for example, in mammalian cells (Xavier *et al*., 2000). Another hypothesis could relate to the scaffolding role of ILK in the integrin complex. We and others have shown that reducing PAT-4/ILK levels in animals from hatching leads to gaps in the cytoskeletal lattice, myofilament detachment, and collapse of MYO-3 in body-wall muscle cells (Meissner *et al*., 2009 and this study). We did not observe such prominent phenotypes in animals in which *pat-4*/ILK was inhibited only during adulthood; however, adult-only inhibition of *pat-4*/ILK could be speculated to cause subtle cytoskeletal disruptions in certain PAT-4/ILK-expressing tissues/cells. In turn, such cytoskeletal disruptions may resemble, and be perceived by HSF-1, as an aggregation-like state. While future experiments are needed to directly investigate these two nonmutually exclusive hypotheses, we have observed that whole-life reduction in *pat-4*/ILK can lead to an early onset of aggregation of polyglutamine-expansion repeats in *C. elegans* (Fig. S7, Supporting information). Moreover, aggregation of human Aβ protein is promoted in *C. elegans* following whole-life inhibition of other integrin-complex components (*i.e*., the integrins *pat-2* and *pat-3*) (Jensen *et al*., 2012). It remains to be directly addressed whether inhibition of *pat-4*/ILK or other integrin-complex components in adult animals could similarly promote a cellular milieu permissive for protein aggregation or misfolding. In such a scenario, activation of HSF-1 could potentially take place via chaperone displacement. For example, Hsp90, which under normal conditions is constitutively bound to HSF-1 to keep HSF-1 in an inactive state, might act as the sensor for subtle cytoskeletal disruptions and dissociate from HSF-1. Interestingly, Hsp90 was recently shown to directly interact with ILK in mammalian cells to facilitate ILK’s interaction with alpha-parvin (Radovanac *et al*., 2013). It remains to be determined whether this interaction between Hsp90 and ILK could represent a functional connection to HSF-1.

In conclusion, we have shown that genetic reduction in the integrin-signaling complex component *pat-4*/ILK activates HSF-1 to induce a neuron-dependent, noncanonical HSR in *C. elegans*. Our study suggests that fine-tuning of such an HSR through neuroendocrine signals can regulate normal physiological processes such as stress responses and aging, and may thus be relevant to ILK-mediated, age-related pathologies such as cancer and cardiomyopathies.

## Experimental procedures

### Strains

Strains were maintained and cultured under standard conditions at 20 °C using *E. coli* OP50 as a food source (Brenner, 1974). For RNAi experiments, animals were grown on HT115 bacteria (see below). For experiments with the temperature-sensitive strains CF512, CF2201 and CF2253, animals were raised at 25 °C until adulthood. See Table S7 (Supporting information) for strains used and created for this study.

### RNA interference (RNAi)

All RNAi bacterial clones (expressing dsRNA for gene of interest) used to feed *C. elegans* for gene inactivation were verified by sequencing and cultured as specified in Supporting Information.

### Pharyngeal pumping

Pharyngeal pumping was measured by counting the number of contractions in the posterior bulb of the pharynx during a 30-s interval. If pharyngeal pumping of a particular animal was irregular, multiple measurements were performed and an average was calculated. Between 20 and 40 animals of each strain were assayed on each day of the experiment, and an average was calculated.

### Lifespan analysis

Lifespan was measured at 20 °C as previously described (Hansen *et al*., 2005), with RNAi treatments initiated as indicated in the text and figure legends. Animals were scored as dead if they failed to respond to gentle prodding with a platinum wire pick. Censoring occurred if animals desiccated on the edge of the plate, escaped, ruptured, or suffered from internal hatching. Statistical analysis was performed using stata software (StataCorp, College Station, TX, USA). *P* values were calculated with the log-rank (Mantel-Cox) method.

### Analysis and imaging of stress reporters

Induction of stress reporters was analyzed by imaging transgenic animals on media plates after anesthetizing the animals with M9 medium containing 0.1% NaN_3_. Images were acquired with a Leica DFC310 FX camera using the exposure times indicated in the figure legends. Image analysis was performed with imagej software (National Institutes of Health, Bethesda, MD, USA), by tracing the intestine and measuring the integrated density of GFP fluorescence.

Imaging of animals with reporter constructs in thermosensory neurons and body-wall muscles was performed at higher magnification using a Zeiss Imager Z1. Fluorescence and differential interference contrast (DIC) microscopy was performed after mounting animals on a 2% agarose pad in M9 medium, both containing 0.1% NaN_3_.

### *C. elegans* and *Drosophila* thermotolerance assays

The survival of *C. elegans* at elevated temperatures was measured after an 8 h incubation at 36 °C (Hansen *et al*., 2007). For each strain, 4 plates with ~20 animals per plate were incubated in a single layer in a HERAtherm incubator (Thermo Fisher, Waltham, MA). Animals were scored as dead if they failed to respond to gentle prodding with a platinum wire pick.

The survival of *Drosophila* at elevated temperatures was measured after an 85 min incubation in a 36 °C waterbath. *ilk*^*54*^ (Zervas *et al*., 2001) was introgressed into the *w*^*CS*^ wild-type background for six generations, and the backcrossed line is referred to as ilk^54-wCS^. Virgin female and male flies were collected and aged at 25 °C. For the thermotolerance assay, 3-week-old flies were briefly anesthetized with CO_2_, separated into groups of 10 flies per food vial, and allowed to recover overnight. The flies were then transferred to empty vials and placed in a waterbath at 36 °C for 85 min. Completely immobile flies were scored as dead.

### Electrophoretic mobility shift assay and Western blot analysis

All biochemical procedures were performed as described in the Supporting Information (Chiang *et al*., 2012).

### Quantitative RT–PCR

Quantitative RT–PCR was performed as previously described (Chiang *et al*., 2012; Lapierre *et al*., 2013). Preparation of *C. elegans* RNA and details of PCR conditions are provided in the Supporting Information. See Table S8 (Supporting information) for primer sequences.

### Statistical analysis

Statistical significance was determined by Student’s t-test and one- or two-way anova using graphpad prism software (La Jolla, CA, USA), except where noted otherwise.

## Supporting Information

**Fig. S1** *pat-4*/ILK mRNA levels are reduced in *C. elegans* by whole-life and adult-only RNAi treatments. **Fig. S2** Effects of reduction of integrin-complex components in *C. elegans* expressing GFP-tagged PAT-4/ILK. **Fig. S3** Effects on stress-inducible GFP reporters following inhibition of *pat-4*/ILK in adult *C. elegans* transcription factor mutants. **Fig. S4** *pat-4*/ILK mRNA levels are reduced in *hsf-1* mutants. **Fig. S5** Reduction of *pat-4*/ILK in *gcy-8* and *ttx-3* mutants does not increase transcription of HSF-1 target genes. **Fig. S6** Thermotactic behavior is intact in *C. elegans* with reduced PAT-4/ILK levels. **Fig. S7** Whole-life inhibition of *pat-4*/ILK increases aggregation of polyglutamine-expansion repeats in *C. elegans*.

**Table S1** Lifespan analysis of various strains subjected to *pat-4/*ILK RNAi. **Table S2** Survival analysis of wild-type animals subjected to RNAi against components of the integrin complex and cytoskeleton during adulthood and incubated at 36°C. **Table S3** Summary of phenotypes observed in animals subjected to RNAi of integrin complex and cytoskeletal components. **Table S4** Survival analysis of transcription factor mutants subjected to *pat-4*/ILK RNAi during adulthood and incubated at 36°C. **Table S5** Survival analysis of mutants with defects in neurons involved in thermosensation subjected to *pat-4*/ILK RNAi during adulthood and incubated at 36°C. **Table S6** Survival analysis of *gon-2* mutants subjected to *pat-4*/ILK RNAi during adulthood and incubated at 36°C. **Table S7** *C. elegans* strains used in this study. **Table S8** Sequences of quantitative RT-PCR primers used in this study.
