# Supplementary figures and images for: Integrin-linked kinase modulates longevity and thermotolerance in C. elegans through neuronal control of HSF-1
Source: Aging Cell. 2014 Jan 9;13(3):419–30. doi: 10.1111/acel.12189 (PMC4059541; doi:10.1111/acel.12189)

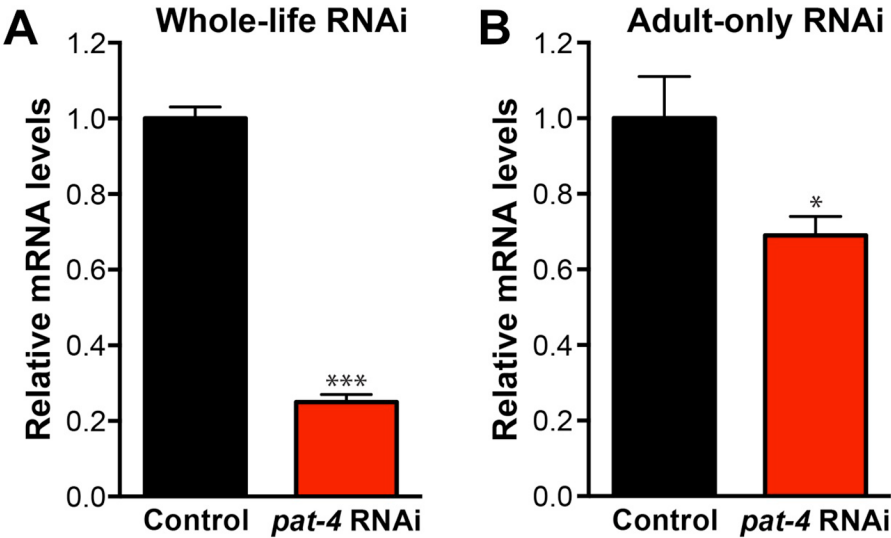

Figure S2

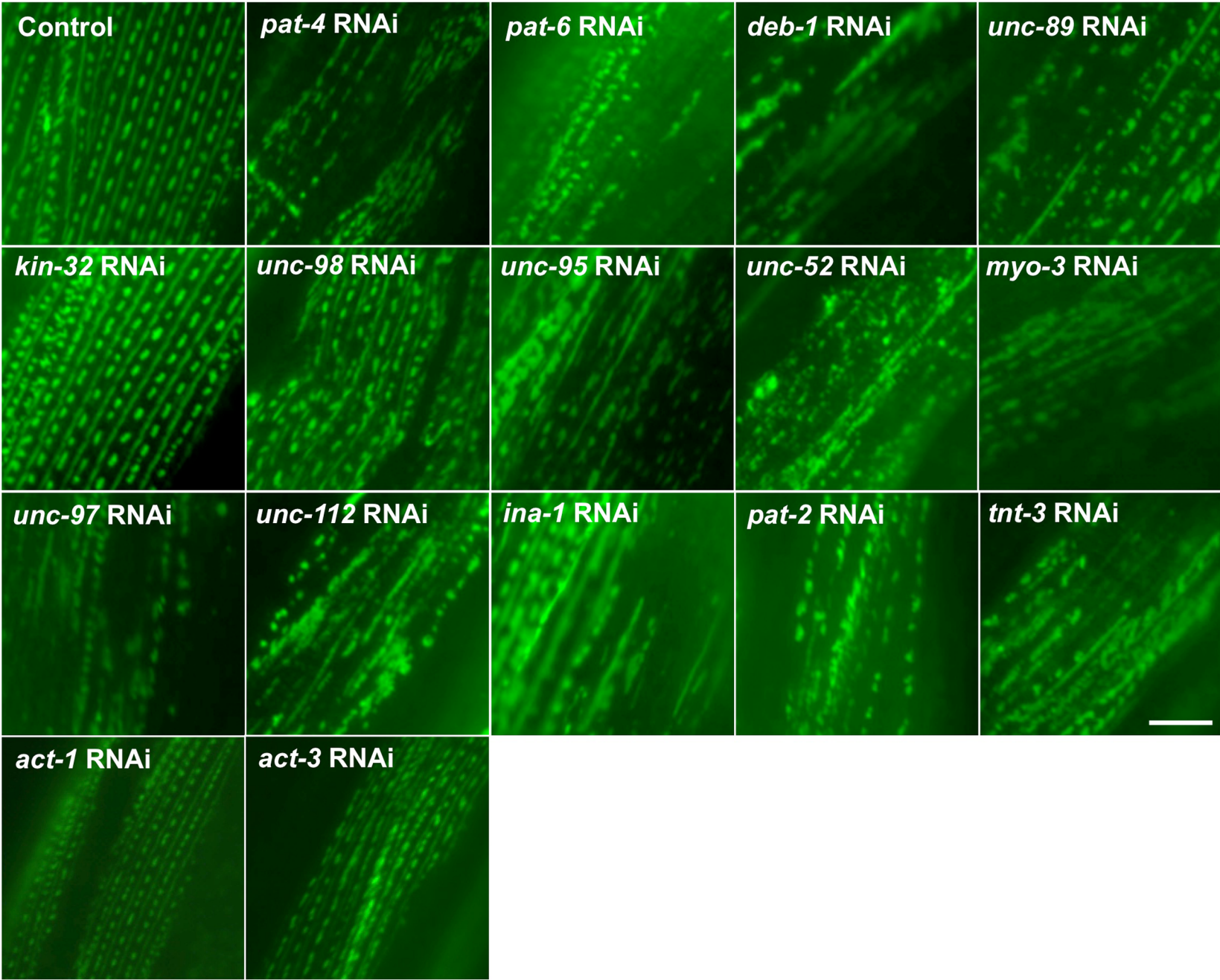

Figure S3

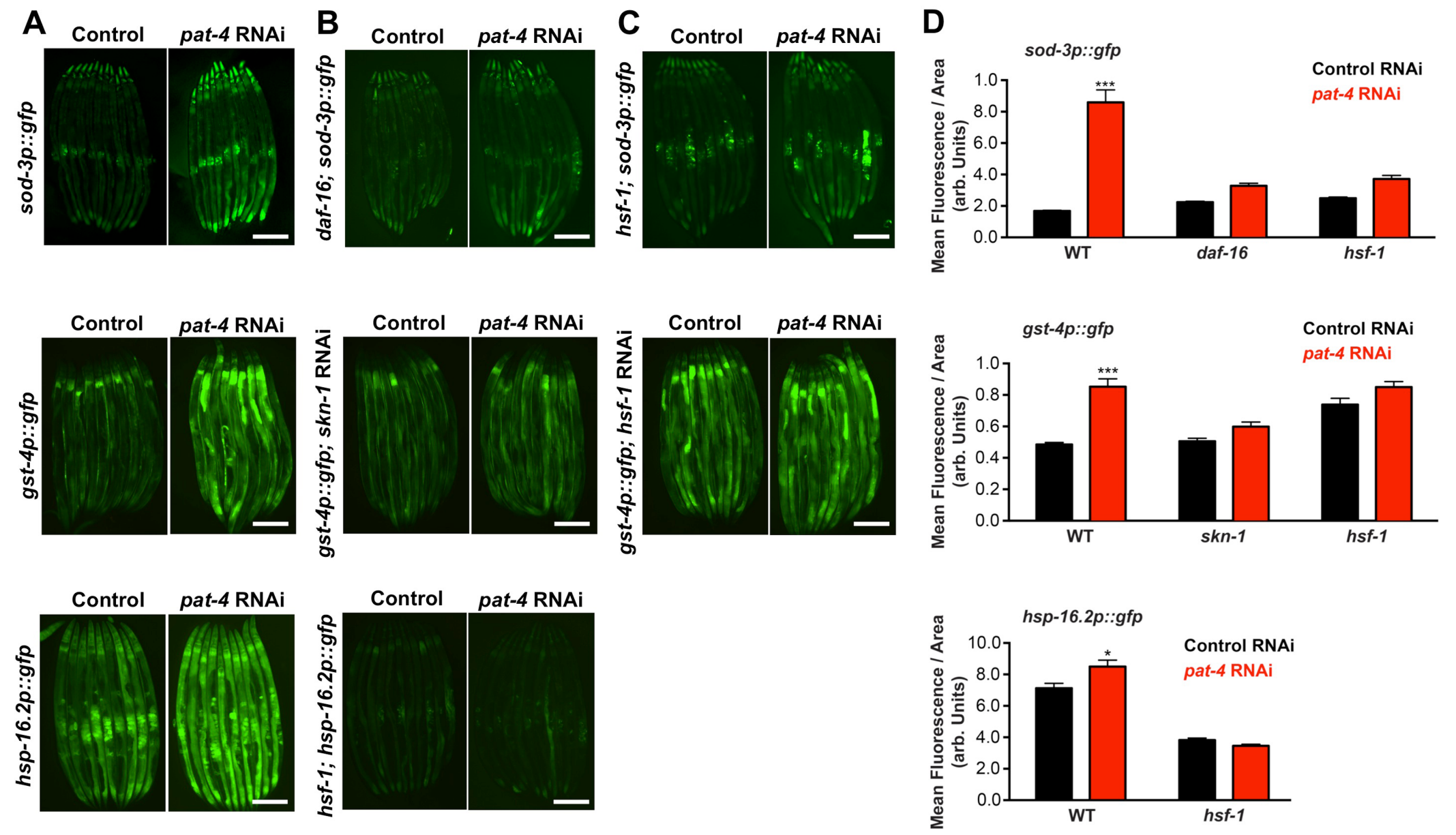

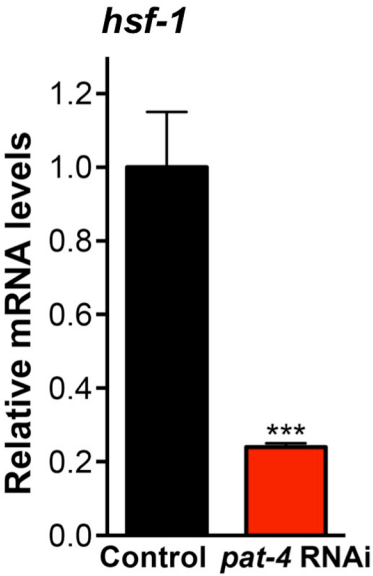

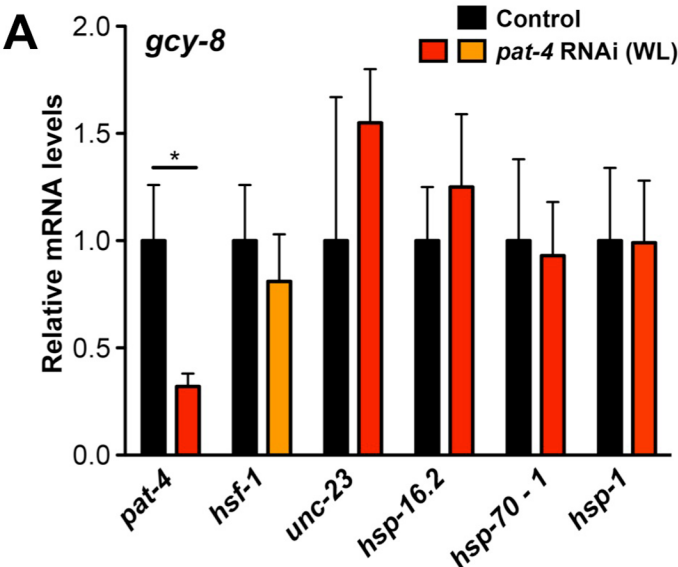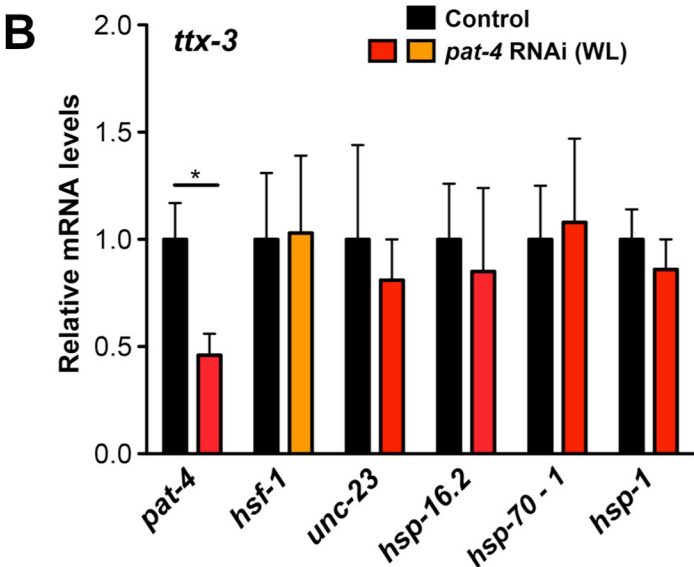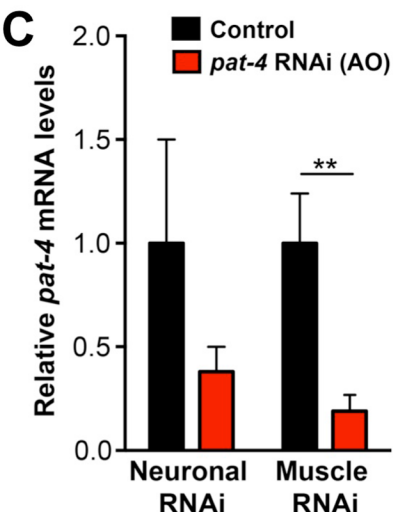

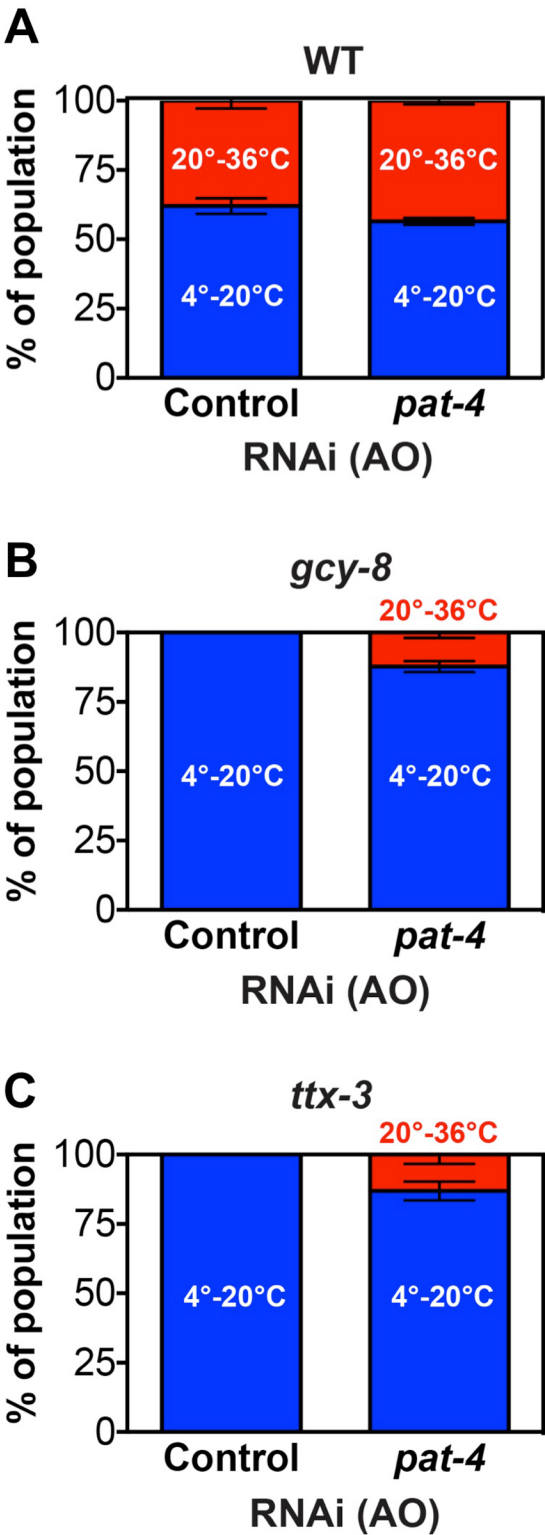

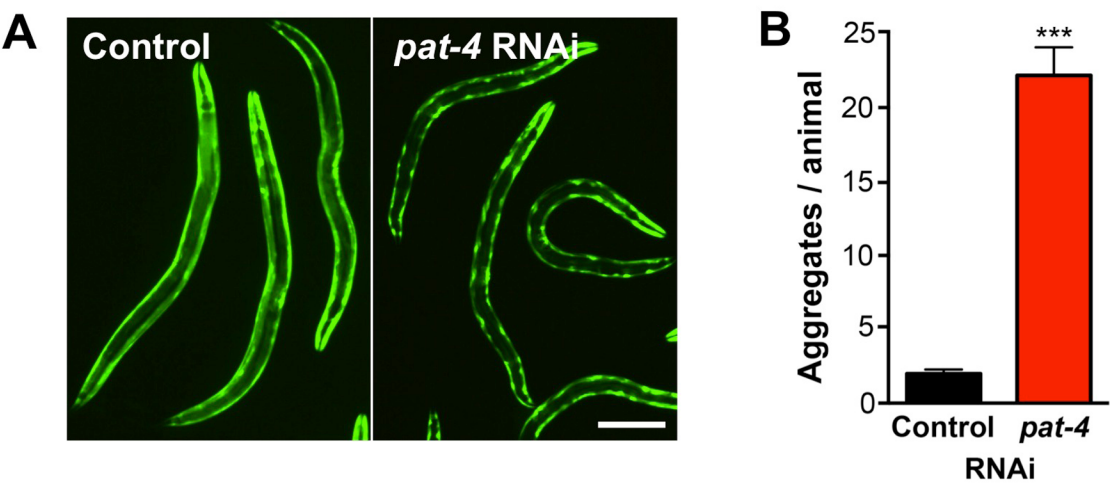

Supplement: Supplementary file 1 — Fig. S1 pat-4/ILK mRNA levels are reduced in C. elegans by whole-life and adult-only RNAi treatments. Fig. S2 Effects of reduction of integrin-complex components in C. elegans expressing GFP-tagged PAT-4/ILK. Fig. S3 Effects on stress-inducible GFP reporters following inhibition of pat-4/ILK in adult C. elegans transcription factor mutants. Fig. S4 pat-4/ILK mRNA levels are reduced in hsf-1 mutants. Fig. S5 Reduction of pat-4/ILK in gcy-8 and ttx-3 mutants does not increase transcription of HSF-1 target genes. Fig. S6 Thermotactic behavior is intact in C. elegans with reduced PAT-4/ILK levels. Fig. S7 Whole-life inhibition of pat-4/ILK increases aggregation of polyglutamine-expansion repeats in C. elegans. [file acel0013-0419-sd1.pdf]
