# Supplementary material for: Integrin-linked kinase modulates longevity and thermotolerance in C. elegans through neuronal control of HSF-1
Source: Aging Cell. 2014 Jan 9;13(3):419–30. doi: 10.1111/acel.12189 (PMC4059541; doi:10.1111/acel.12189)
Supplement: Supplementary file 2 — Table S1 Lifespan analysis of various strains subjected to pat-4/ILK RNAi. Table S2 Survival analysis of wild-type animals subjected to RNAi against components of the integrin complex and cytoskeleton during adulthood and incubated at 36°C. Table S3 Summary of phenotypes observed in animals subjected to RNAi of integrin complex and cytoskeletal components. Table S4 Survival analysis of transcription factor mutants subjected to pat-4/ILK RNAi during adulthood and incubated at 36°C. Table S5 Survival analysis of mutants with defects in neurons involved in thermosensation subjected to pat-4/ILK RNAi during adulthood and incubated at 36°C. Table S6 Survival analysis of gon-2 mutants subjected to pat-4/ILK RNAi during adulthood and incubated at 36°C. Table S7 C. elegans strains used in this study. Table S8 Sequences of quantitative RT-PCR primers used in this study. [file acel0013-0419-sd2.doc]

## SUPPORTING INFORMATION

**Integrin-linked kinase modulates longevity and thermotolerance in *C. elegans* through neuronal control of HSF-1**

Caroline Kumsta, Tsui-Ting Ching, Mayuko Nishimura, Andrew E. Davis, Sara Gelino, Hannah H. Catan, Xiaokun Yu, Chu-Chiao Chu, Binnan Ong, Siler H. Panowski, Nathan Baird, Rolf Bodmer, Ao-Lin Hsu, and Malene Hansen

**Supporting Information Inventory:**

**1. Supporting Experimental Procedures**

**2. Supporting References**

**3. Supporting Tables**

**4. Supporting Figure Legends**

**SUPPORTING EXPERIMENTAL PROCEDURES**

**RNAi clones and treatments**

Control HT115 bacteria either contained empty vector (L4440) or expressed dsRNA against *gfp* (gift from Andrew Dillin)*.* The following RNAi bacterial clones were obtained from the Ahringer library (JA) , the Vidal RNAi library (MV) , or the Dillin lab (AD) : *hsf-1* (JA)*, pha-4* (JA)*, skn-1* (JA), *daf-16* (AD), *daf-2* (AD), *pat-4* (JA)*, pat-6* (JA)*, deb-1* (MV)*, unc-89* (JA)*, unc-98* (MV)*, unc-95* (MV)*, unc-52* (JA)*, unc-97* (JA)*, unc-112* (JA)*, ina-1* (JA)*, pat-2* (JA)*, tnt-3* (JA)*, act-1* (JA), *act-3* (JA), and *myo-3* (JA). To create an RNAi clone for FAK/Focal Adhesion Kinase, we amplified a 534 bp fragment of *kin-32* from genomic DNA and cloned it into the L4440 vector using *Nhe* I and *Xho* I restriction sites. Primer sequences used for amplification are available upon request.

For RNAi treatments HT115 bacteria were grown in LB liquid culture containing 0.1 mg/ml carbenicillin (BioPioneer, San Diego, CA), and an 80 μl aliquot was spotted onto a 6 cm NGM plate. Bacteria were allowed to grow for 1–2 days and then eggs or animals were transferred onto plates with the appropriate dsRNA-expressing or control bacteria. For induction of dsRNA expression, 80 μl of a solution containing 0.1 M IPTG (Promega, Madison, WI) and 50 µg/ml carbenicillin was placed directly onto the lawn. For whole-life RNAi, animals were synchronized by hypochlorous acid treatment and the eggs were transferred directly to NGM plates seeded with dsRNA-expressing or control bacteria. For adult-only RNAi, animals were synchronized by hypochlorous acid treatment and the eggs were first transferred to NGM plates seeded with OP50 bacteria and then transferred on day 1 of adulthood to NGM plates seeded with dsRNA-expressing or control bacteria.

**Constructs and transgenic animals**

To construct plasmids expressing *sid-1* cDNA driven by various promoters, full-length *sid-1* cDNA (2330 bp) was cloned from first-strand worm cDNA by PCR amplification and inserted in the *C. elegans* expression vector pPD95.77 using *Xma I* and *Age* I restriction enzymes. The *unc-54* 3’ UTR was PCR amplified with a 5’ *Age I* site and a 3’ *BsiW I* site and cloned into the *sid-1*/pPD95.77 vector in the place of the *gfp::unc-54* 3’ UTR fragment.

To create the body-wall-muscle expression construct, the *myo-3* (2385 bp) promoter was cloned in front of the *sid-1* cDNA using *Afl II* 5' and *Age I* 3' following digestion of the *sid-1*/pPD95.77 vector with *Afl II* and *Xma I.* As co-injection marker for the muscle expression vector, td*tomato* was amplified from vector s260(pPD97.77/td*tomato*) (the original td*tomato* sequence (in pCMVtd*tomato*) was obtained as a gift from Roger Tsien) and cloned into the *myo-3p*::*sid-1*/pPD95.77 via *Xma I/Age I* digest and thus replaced *sid-1*. To create the neuronal expression construct, the *rab-3* (4919 bp) promoter was cloned in front of the *sid-1* cDNA using *Sph I* and *Xma I* restriction enzymes. As co-injection marker for the neuronal expression strain, td*tomato* was amplified from vector s260(pPD97.77/td*tomato*) and cloned into the *C. elegans* expression vector pPD95.77 using *Age I* and *Bsm I*, followed by cloning in the *rab-3p* promoter sequence using *Sph I* and *Xma I.*

The *sid-1*-containing vectors, in addition to the respective td*tomato-*containing co-injection marker were microinjected into the gonads of adult *eat-2(ad1116); sid-1(qt9)* hermaphrodite animals by using standard methods at a concentration of 10 ng/ul. All DNA mixes for injection were brought to a final total concentration of 100 ng/ul using pPD61.125 as “Filler” DNA. F1 progeny were selected on the basis of the marker phenotype. Individual F2 worms were isolated to establish independent lines.

Extrachromosomal arrays were integrated as described and these strains were outcrossed to *sid-1(qt9)* (which caused elimination of the *eat-2(ad1116)* mutation).

**Quantitative RT-PCR**

Total RNA was isolated from a synchronized population of ~2000 nematodes on day 2 of adulthood after whole-life RNAi treatment or day 3 of adulthood after adult-only RNAi treatment. After harvest, the animals were flash frozen in liquid nitrogen. RNA was extracted with Trizol (Life Technologies, Carlsbad, CA) and purified using a Qiagen RNeasy kit, with an additional DNA digestion step (Qiagen DNase I kit, Venlo, Netherlands). Reverse transcription (1 µg per sample) was performed using M-MuLV reverse transcriptase and random 9-mer primers (New England Biolabs, Ipswich, MA) . Quantitative PCR was performed using SYBR Green Master Mix in an LC480 LightCycler (Roche, Basel, Switzerland). A standard curve was obtained for each primer set by serially diluting a mixture of different cDNAs, and the standard curves were used to convert the observed CT values to relative values. mRNA levels of target genes were normalized against the mean of two to four of the following housekeeping genes and are specified in the figure legends: *ama-1* (large subunit of RNA polymerase II), nuclear hormone receptor *nhr-23*, the Rho-GTPase *cdc-42*, and the putative ABC transporter *pmp-3* . Primer sequences for HSF-1 target genes were obtained from published sources ; and are listed in **Table S8**. The *hsf-1* target gene *unc-23* (sequence H14N18.1) was previously published under the gene name *unc-33* . Each biological sample was analyzed with three technical replicates. The average and standard error of the mean of every mRNA was calculated and compared by one-way ANOVA or Student’s *t*-test (GraphPad Prism, La Jolla, CA).

**Preparation of nuclear extracts**

Animals were raised on bacteria expressing dsRNA for *pat-4*/ILK or control bacteria and harvested at day 1 of adulthood. Nematode pellets were homogenized in an equal volume of 2X NPB buffer (20 mM HEPES, pH 7.6, 20 mM KCl, 3 mM MgCl_2_, 2mM EDTA, 0.5 M sucrose, 1 mM dithiothreitol, protease inhibitors, and phosphatase inhibitors) using a Kontes Pellet Pestle tissue grinder. The suspension was centrifuged (4000 g, 5 min, 4ºC) and the pellets were further homogenized by 20 strokes with pestle A of a Dounce homogenizer. The pellet was resuspended in NPB buffer with 0.25% NP-40 and 0.1% Triton-X100, recentrifuged, and washed three times with the same buffer. The nuclear pellet was extracted with four volumes of HEG buffer (20 mM HEPES, pH 7.9, 0.5 mM EDTA, 10% glycerol, 0.42 M NaCl, 1.5 mM MgCl_2_, and protease inhibitors) at 4ºC for 45 min. Finally, the nuclear fraction was collected by centrifugation at 14,000 g for 15 min at 4°C. Protein concentrations were determined with a Bradford assay kit (Bio-Rad, Hercules, CA).

**Electrophoretic mobility shift assay (EMSA)**

EMSA was carried out as previous described using *C. elegans* nuclear extracts prepared as described above. In brief, 1 µg of extract was mixed with 1 mg/mL of poly (dI•dC) and 1 nM of a biotin-labeled oligonucleotide containing the heat-shock element (HSE) sequence of *hsp-16.1* , and incubated for 15 min at room temperature in binding buffer [20 mM HEPES, pH 7.6, 5 mM EDTA, 1 mM dithiothreitol, 150 mM KCl, 50 mM (NH_4_)_2_SO_4_, and 1% Tween 20 (v/v)]. After incubation, the samples were separated by native 3.5% PAGE and the HSF-1–HSE DNA complexes were visualized using a LightShift Chemiluminescent EMSA kit (Pierce, Rockford, IL). The bands were quantified by densitometry using Image J software (National Institute of Health). Statistical analysis was performed using GraphPad Prism 5.0 (GraphPad, La Jolla, CA).

**Western blot analysis**

Total protein extracts were prepared from 200–500 synchronized adult nematodes grown on dsRNA-expressing or control bacteria at 20ºC. Animals were harvested by washing three times with cold M9 buffer and centrifuged. Pellets were resuspended in 5–10 volumes of SDS sample buffer and boiled for 5 min. The samples were then resolved by SDS-PAGE and proteins were transferred to a PVDF membrane (Millipore, Billerica, MA). The membrane was incubated with anti-HSF1 antibody (1:2000) (Abcam, Cambridge, England #Ab2923) at 4°C for 12 h, washed three times with TBST, and then incubated with HRP-conjugated anti rabbit IgG antibody for 1 h at room temperature. The membrane was washed again three times with TBST and incubated with a chemiluminescent substrate (Pierce, Rockford, IL). Bands were visualized by autoradiography and quantified by densitometry using Image J software (National Institute of Health).

**Thermorecovery assay**

Survival at elevated temperatures of *gon-2(q388)* mutants was measured in thermotolerance (see main text) or thermorecovery assays. For the latter, *C. elegans* was subjected to RNAi treatment as indicated in the text and incubated at 6 h at 36°C in a HERAtherm incubator (ThermoFisher, Waltham, MA), followed by a ‘recovery’ period of 20 h at 20°C . Survival was subsequently assessed by scoring voluntary movement of animals.

**Thermotactic movement assay**

NGM plates with OP50 bacteria were incubated at 4°C or 36°C for 3-4 hours. Chunks (~0.8 cm^3^) were then excised from the plates and placed (bacterial surface face down) onto a fresh plate lacking bacteria, at equal distances from the center of the plate. Wild-type *C. elegans, gcy-8(oy44)* mutants, and *ttx-3(ks5)* mutants were washed and starved in liquid M9 for 20 min. Animals (12-20) were then spotted onto the center of the NGM plates, and the animals were allowed to migrate toward the food for 20 min. The number of animals in the chunks prepared at 4°C or 36°C was then counted.

**SUPPORTING REFERENCES**

Chiang WC, Ching TT, Lee HC, Mousigian C , Hsu AL (2012). HSF-1 regulators DDL-1/2 link insulin-like signaling to heat-shock responses and modulation of longevity. *Cell*. **148**, 322-334.

Dillin A, Crawford DK , Kenyon C (2002). Timing requirements for insulin/IGF-1 signaling in C. elegans. *Science*. **298**, 830-834.

GuhaThakurta D, Palomar L, Stormo GD, Tedesco P, Johnson TE, Walker DW, Lithgow G, Kim S , Link CD (2002). Identification of a novel cis-regulatory element involved in the heat shock response in Caenorhabditis elegans using microarray gene expression and computational methods. *Genome research*. **12**, 701-712.

Hansen M, Taubert S, Crawford D, Libina N, Lee SJ , Kenyon C (2007). Lifespan extension by conditions that inhibit translation in Caenorhabditis elegans. *Aging Cell*. **6**, 95-110.

Hoogewijs D, Houthoofd K, Matthijssens F, Vandesompele J , Vanfleteren JR (2008). Selection and validation of a set of reliable reference genes for quantitative sod gene expression analysis in C. elegans. *BMC Mol Biol*. **9**, 9.

Hope IA (1999). In *C. elegans - A practical approach*. Oxford, UK: Oxford University Press, pp. 62-63.

Hsu AL, Murphy CT , Kenyon C (2003). Regulation of aging and age-related disease by DAF-16 and heat-shock factor. *Science*. **300**, 1142-1145.

Kamath RS , Ahringer J (2003). Genome-wide RNAi screening in Caenorhabditis elegans. *Methods*. **30**, 313-321.

Kumsta C , Hansen M (2012). C. elegans rrf-1 Mutations Maintain RNAi Efficiency in the Soma in Addition to the Germline. *PLoS One*. **7**, e35428.

Lapierre LR, De Magalhaes Filho CD, McQuary PR, Chu CC, Visvikis O, Chang JT, Gelino S, Ong B, Davis AE, Irazoqui JE, Dillin A , Hansen M (2013). The TFEB orthologue HLH-30 regulates autophagy and modulates longevity in Caenorhabditis elegans. *Nat Commun*. **4**, 2267.

Mackinnon AC, Qadota H, Norman KR, Moerman DG , Williams BD (2002). C. elegans PAT-4/ILK functions as an adaptor protein within integrin adhesion complexes. *Current biology : CB*. **12**, 787-797.

Mello CF, A. (1995). DNA Transformation. In *Methods in Cell Biology*): Academic Press, Inc., pp. 451-482.

Prahlad V, Cornelius T , Morimoto RI (2008). Regulation of the cellular heat shock response in Caenorhabditis elegans by thermosensory neurons. *Science*. **320**, 811-814.

Rual JF, Ceron J, Koreth J, Hao T, Nicot AS, Hirozane-Kishikawa T, Vandenhaute J, Orkin SH, Hill DE, van den Heuvel S , Vidal M (2004). Toward improving Caenorhabditis elegans phenome mapping with an ORFeome-based RNAi library. *Genome Res*. **14**, 2162-2168.

Sun AY , Lambie EJ (1997). gon-2, a gene required for gonadogenesis in Caenorhabditis elegans. *Genetics*. **147**, 1077-1089.

Taubert S, Van Gilst MR, Hansen M , Yamamoto KR (2006). A Mediator subunit, MDT-15, integrates regulation of fatty acid metabolism by NHR-49-dependent and -independent pathways in C. elegans. *Genes Dev*. **20**, 1137-1149.

**SUPPORTING TABLES**

**Table S1. Lifespan analysis of various strains subjected to *pat-4/*ILK** RNAi.

| **Exp** | **Strain** | **RNAi** | **MLS/days** | **N** | **Control** | | **% MLS** | **P value** |
| --- | --- | --- | --- | --- | --- | --- | --- | --- |
|  |  |  |  |  | **MLS/days** | **N** |  |  |
| **Adult-only RNAi treatment** | | | | | | | | |
| 1^1^ | WT | *pat-4* | 20.2 | 83/97 | 16.8 | 87/100 | + 16.8% | 0.0001 (***) |
| 2 | WT | *pat-4* | 21.3 | 63/90 | 18.5 | 67/90 | + 15.1% | 0.0050 (**) |
| 3 | WT | *pat-4* | 19.4 | 80/95 | 16.5 | 73/96 | + 17.5% | 0.0014 (**) |
| 4 | WT | *pat-4* | 18.0 | 93/109 | 16 | 96/106 | + 12.5% | 0.018 (*) |
| 5 | WT | *pat-4* | 16.8 | 90/106 | 14.3 | 58/95 | + 17.5% | 0.0098 (**) |
| 1^2^ | WT | *pat-4* | 20.2 | 84/101 | 15.9 | 86/99 | + 27.0% | <0.0001 (***) |
|  | *hsf-1* | *pat-4* | 13.6 | 93/95 | 13.4 | 85/105 | + 1.5% | 0.81 (n.s.) |
| 2 | WT | *pat-4* | 17.9 | 93/100 | 14.9 | 98/102 | + 20.1% | <0.0001 (***) |
|  | *hsf-1* | *pat-4* | 13.3 | 59/102 | 13.5 | 64/100 | - 1.5% | 0.53 (n.s.) |
| 3 | WT | *pat-4* | 20.5 | 104/123 | 19.0 | 100/120 | + 7.9% | 0.023 (*) |
|  | *hsf-1* | *pat-4* | 11.4 | 89/113 | 14.1 | 102/115 | - 19.1% | 0.0012 (**) |
| 4 | WT | *daf-2* | 30.3 | 86/110 | 17.6 | 92/110 | + 72.2% | <0.0001 (***) |
|  | *hsf-1* | *daf-2* | 18.6 | 27/91 | 15.7 | 28/97 | + 18.5% | 0.017 (*) |
| 1^3^ | WT | *pat-4* | 23.8 | 69/102 | 17.9 | 83/102 | + 22.2% | <0.0001 (***) |
|  | *gcy-8* | *pat-4* | 13.1 | 94/102 | 12.8 | 89/102 | - 27.8% | 0.84 (n.s.) |
|  | *ttx-3* | *pat-4* | 16.1 | 98/102 | 18.0 | 93/102 | - 16.7% | 0.12 (n.s.) |
| 2 | WT | *pat-4* | 16.8 | 92/108 | 15.5 | 100/108 | + 8.3% | 0.053 (n.s.) |
|  | *gcy-8* | *pat-4* | 13.9 | 88/109 | 13.6 | 100/110 | + 2.2% | 0.65 (n.s.) |
|  | *ttx-3* | *pat-4* | 16.3 | 88/108 | 15.8 | 109/114 | + 3.1% | 0.33 (n.s.) |
| 3 | WT | *pat-4* | 24.5 | 88/108 | 21.4 | 76/108 | + 14.3% | 0.0003 (***) |
|  | *gcy-8* | *pat-4* | 15.9 | 107/108 | 15.3 | 94/108 | + 3.9% | 0.76 (n.s.) |
|  | *ttx-3* | *pat-4* | 18.5 | 95/108 | 20.5 | 82/108 | - 9.8% | 0.017 (*) |
| 1^4^ | WT | *pat-4* | 21.9 | 67/111 | 16.3 | 92/110 | + 34.4% | <0.0001(***) |
|  | *rab-3p ::sid-1* | *pat-4* | 21.4 | 92/118 | 16.2 | 104/116 | + 29.6% | <0.0001 (***) |
|  | *myo-3p ::sid-1* | *pat-4* | 15.7 | 117/118 | 16.8 | 89/116 | - 6.5% | 0.036 (*) |

**Table S1 (continued).**

| **Exp** | **Strain** | **RNAi** | **MLS/days** | **N** | **Control** | | **% MLS** | **P value** |
| --- | --- | --- | --- | --- | --- | --- | --- | --- |
|  |  |  |  |  | **MLS/days** | **N** |  |  |
| **Adult-only RNAi treatment** | | | | | | | | |
| 2 | WT | *pat-4* | 18.5 | 75/122 | 14.5 | 75/122 | + 27.6% | 0.0002 (***) |
|  | *rab-3p ::sid-1* | *pat-4* | 18.6 | 88/109 | 16.6 | 94/116 | + 12.0% | 0.021 (*) |
|  | *myo-3p ::sid-1* | *pat-4* | 12.8 | 92/116 | 15.5 | 80/108 | - 17.4% | 0.0011 (**) |
| 3 | WT | *pat-4* | 18.6 | 85/116 | 16.0 | 99/116 | + 16.2% | 0.011 (*) |
|  | *rab-3p ::sid-1* | *pat-4* | 23.1 | 96/120 | 18.9 | 111/118 | + 22.2% | <0.0001 (***) |
|  | *myo-3p ::sid-1* | *pat-4* | 14.3 | 105/118 | 17.2 | 104/113 | - 16.9% | 0.0003 (***) |
| **Whole-life RNAi treatment** | | | | | | | | |
| 1 | WT | *pat-4* | 22.5 | 36/113 | 18.5 | 89/102 | + 21.6% | 0.0010 (**) |
|  |  | *daf-2* | 27.9 | 70/109 | 18.5 | 89/102 | + 50.8% | <0.0001 (***) |
|  | *hsf-1* | *pat-4* | 15.6 | 85/109 | 15.9 | 85/103 | - 1.3% | 0.94 (n.s.) |
|  |  | *daf-2* | 21.8 | 36/90 | 15.9 | 85/103 | + 38.0% | <0.0001 (***) |
| 2 | WT | *pat-4* | 19.2 | 122/127 | 15.5 | 93/98 | + 23.9% | <0.0001 (***) |
|  | *hsf-1* | *pat-4* | 13.8 | 44/100 | 14.8 | 103/107 | - 6.8% | 0.27 (n.s.) |
| 3 | WT | *pat-4* | 21.6 | 90/114 | 16.6 | 93/111 | + 30.1% | <0.0001 (***) |
|  | *hsf-1* | *pat-4* | 13.9 | 80/110 | 13.4 | 116/129 | + 3.7% | 0.27 (n.s.) |
| 1 | WT | *pat-4* | 22.7 | 100/108 | 19.4 | 95/108 | + 17.0% | <0.0001 (***) |
|  | *gcy-8* | *pat-4* | 14.7 | 102/108 | 16.3 | 97/108 | - 9.8% | 0.17 (n.s.) |
|  | *ttx-3* | *pat-4* | 16.1 | 63/108 | 17.7 | 97/108 | - 10.0% | 0.12 (n.s.) |
| 2 | WT | *pat-4* | 22.3 | 113/118 | 17.6 | 101/108 | + 26.7% | <0.0001 (***) |
|  | *gcy-8* | *pat-4* | 13.0 | 115/117 | 11.2 | 102/112 | + 16.1% | 0.0017 (*) |
|  | *ttx-3* | *pat-4* | 15.3 | 84/119 | 14.1 | 101/112 | + 8.5% | 0.58 (n.s.) |

**Table S1.** Lifespan analysis of wild-type (WT, N2), *hsf-1(sy441)*, *gcy-8(oy44),* *ttx-3(ks5)* animals and tissue-specific RNAi strains (neurons: *sid-1(qt9); rab-3p::sid-1*, muscles: *sid-1(qt9); myo-3p::sid-1*, listed in table by transgene only, see **Table S7** for full genotype) fed control bacteria or bacteria expressing dsRNA against *pat-4*/ILK. As control, bacteria expressing either empty vector or dsRNA against *gfp* were used [empty-vector and *gfp* RNAi clones have equivalent effects on lifespan (data not shown)].

For adult-only RNAi experiments (upper section), the animals were raised on OP50 bacteria until they reached day 1 of adulthood and were then moved to plates containing dsRNA-expressing bacteria. For whole-life RNAi experiments (lower section), animals were fed dsRNA-expressing bacteria from hatching. The experiment number for each strain is shown in the first column.

MLS, mean lifespan. N*,* observed deaths/total number of animals subjected to RNAi. % MLS, % lifespan extension compared to control. *P* values (log-rank test): n.s., not significant (*P*> 0.05), **P <*0.05, ***P* < 0.01, ****P* < 0.001. Superscript numbers denote the experiments shown in the following figures: ^1^ **Fig. 1I**, ^2^ **Fig. 2H**, ^3^ **Fig. 4C–D,** ^4^ **Fig. 5C–D**.

**Table S2. Survival analysis of wild-type animals subjected to RNAi against components of the integrin complex and cytoskeleton during adulthood and incubated at 36°**C.

| **RNAi targeting integrin complex and cytoskeletal components** | | | | | **Control RNAi** | | ***pat-4*/ILK RNAi** | | |
| --- | --- | --- | --- | --- | --- | --- | --- | --- | --- |
| **RNAi** | **Exp** | **Survival  (% alive  ± SEM)** | **N** | **P value**  **(compared to Control RNAi)** | **Survival  (% alive  ± SEM)** | **N** | **Survival  (% alive  ± SEM)** | **N** | **P value**  **(compared to Control RNAi)** |
| ***pat-6*** | 1 | 66±1 | 82 | 0.0005 (***) | 27±4 | 84 | 65±5 | 71 | 0.001 (**) |
| **(Parvin/ Acto-paxin)** | 2 | 40±5 | 63 | 0.0008 (***) | 4±3 | 66 | 22±4 | 55 | 0.01 (*) |
|  | 3 | 76±3 | 71 | 0.002 (**) | 42±6 | 71 | 69±3 | 77 | 0.007 (**) |
|  | 4 | 28±2 | 89 | 0.009 (**) | 11±4 | 80 | 40±4 | 69 | 0.002 (**) |
|  | 5 | 59±3 | 112 | 0.02 (*) | 44±4 | 114 | 64±4 | 112 | 0.01 (*) |
| ***deb-1*** | 1 | 34±2 | 66 | 0.0002 (***) | 4±3 | 66 | 22±4 | 55 | 0.01 (*) |
| **(Vinculin**) | 2 | 67±4 | 112 | 0.007 (**) | 44±4 | 114 | 64±4 | 112 | 0.01 (*) |
|  | 4 | 54±3 | 80 | 0.002 (**) | 28±4 | 96 | 58±6 | 79 | 0.006 (**) |
|  | 3 | 64±2 | 72 | 0.01 (*) | 41±6 | 65 | 71±4 | 63 | 0.006 (**) |
|  | 5 | 18±4 | 68 | 0.01 (*) | 2±2 | 67 | n.a. | n.a. | n.a. |
| ***unc-89*** | 1 | 27±2 | 68 | 0.0007 (***) | 4±3 | 66 | 22±4 | 55 | 0.01 (*) |
| **(Obscurin)** | 2 | 27±4 | 68 | 0.001 (**) | 2±2 | 67 | n.a. | n.a. | n.a. |
|  | 3 | 58±3 | 72 | 0.02 (*) | 31±8 | 74 | 81±4 | 64 | 0.001 (**) |
|  | 4 | 20±4 | 56 | 0.3 (n.s.) | 14±4 | 57 | 41±4 | 75 | 0.003 (**) |
| ***kin-32*** | 1 | 65±4 | 93 | 0.0006 (***) | 28±4 | 96 | 58±6 | 79 | 0.006 (**) |
| **(Focal adhesion kinase)** | 2 | 50±10 | 75 | 0.005 (**) | 4±3 | 66 | 22±4 | 55 | 0.01 (*) |
|  | 3 | 42±4 | 72 | 0.9 (n.s.) | 41±6 | 65 | 71±4 | 63 | 0.006 (**) |
| ***unc-98*** | 1 | 57±2 | 78 | 0.0005 (***) | 27±4 | 84 | 65±5 | 71 | 0.001 (**) |
|  | 2 | 46±2 | 85 | 0.007 (**) | 28±4 | 96 | 58±6 | 79 | 0.006 (**) |
|  | 3 | 44±7 | 61 | 0.3 (n.s) | 33±5 | 65 | 57±5 | 58 | 0.02 (*) |
|  | 4 | 21±3 | 78 | 0.2 (n.s.) | 15±3 | 77 | 46±6 | 64 | 0.004 (**) |

Table S2 (continued).

| **RNAi targeting integrin complex and cytoskeletal components** | | | | | **Control RNAi** | | ***pat-4*/ILK RNAi** | | |
| --- | --- | --- | --- | --- | --- | --- | --- | --- | --- |
| **RNAi** | **Exp** | **Survival  (% alive  ± SEM)** | **N** | **P value**  **(compared to Control RNAi)** | **Survival  (% alive  ± SEM)** | **N** | **Survival  (% alive  ± SEM)** | **N** | **P value**  **(compared to Control RNAi)** |
| ***unc-95*** | 1 | 30±4 | 68 | 0.0008 (***) | 2±2 | 67 | n.a. | n.a. | n.a. |
| **(Paxillin)** | 2 | 50±5 | 79 | 0.01 (*) | 28±4 | 96 | 58±6 | 79 | 0.006 (**) |
|  | 3 | 15±4 | 75 | 0.07 (n.s.) | 4±3 | 66 | 22±4 | 55 | 0.01 (*) |
|  | 4 | 46±3 | 67 | 0.5 (n.s.) | 41±6 | 65 | 71±4 | 63 | 0.006 (**) |
| ***unc-52*** | 1 | 42±4 | 80 | 0.0005 (***) | 27±4 | 84 | 65±5 | 71 | 0.001 (**) |
| **(Perlecan)** | 2 | 16±6 | 42 | 0.8 (n.s.) | 14±4 | 57 | 41±4 | 75 | 0.003 (**) |
|  | 3 | 23±5 | 75 | 0.2 (n.s.) | 15±3 | 77 | 46±6 | 64 | 0.004 (**) |
| ***myo-3*** | 1 | 58±2 | 77 | 0.05 (*) | 42±6 | 71 | 69±3 | 77 | 0.007 (**) |
| **(Myosin heavy** **chain)** | 2 | 37±6 | 63 | 0.6 (n.s.) | 31±8 | 74 | 81±4 | 64 | 0.001 (**) |
|  | 3 | 24±4 | 70 | 0.6 (n.s.) | 27±4 | 84 | 65±5 | 71 | 0.001 (**) |
| ***unc-97*** | 1 | 29±3 | 80 | 0.001 (**) | 4±3 | 66 | 22±4 | 55 | 0.01 (*) |
| **(Pinch)** | 2 | 44±9 | 61 | 0.3 (n.s.) | 33±5 | 65 | 57±5 | 58 | 0.02 (*) |
|  | 3 | 10±1 | 64 | 0.4 (n.s.) | 14±4 | 57 | 41±4 | 75 | 0.003 (**) |
|  | 4 | 18±3 | 57 | 0.5 (n.s.) | 15±3 | 77 | 46±6 | 64 | 0.004 (**) |
|  | 5 | 0±0 | 67 | 0.4 (n.s.) | 2±2 | 67 | n.a. | n.a. | n.a. |
| ***unc-112*** | 1 | 23±9 | 52 | 0.9 (n.s.) | 14±4 | 57 | 41±4 | 75 | 0.003 (**) |
| **(Kindlin)** | 2 | 23±3 | 74 | 0.1 (n.s) | 15±3 | 77 | 46±6 | 64 | 0.004 (**) |
|  | 3 | 12±5 | 70 | 0.2 (n.s.) | 4±3 | 66 | 22±4 | 55 | 0.01 (*) |
| ***ina-1*** | 1 | 8±1 | 65 | 0.5 (n.s) | 11±4 | 80 | 40±4 | 69 | 0.002 (**) |
| **(Alpha-integrin)** | 2 | 11±3 | 59 | 0.2 (n.s.) | 4±3 | 66 | 22±4 | 55 | 0.01 (*) |
|  | 3 | 3±2 | 70 | 0.7 (n.s.) | 2±2 | 67 | n.a. | n.a. | n.a. |
| ***pat-2*** | 1 | 28±7 | 75 | 0.08 (n.s.) | 11±4 | 80 | 40±4 | 69 | 0.002 (**) |
| **(Alpha-integrin)** | 2 | 42±11 | 46 | 0.2 (n.s) | 22±5 | 45 | 49±3 | 47 | 0.004 (**) |
|  | 3 | 13±5 | 63 | 0.09 (n.s.) | 2±2 | 67 | n.a. | n.a. | n.a. |

Table S2 (continued).

| **RNAi targeting integrin complex and cytoskeletal components** | | | | | **Control RNAi** | | ***pat-4*/ILK RNAi** | | |
| --- | --- | --- | --- | --- | --- | --- | --- | --- | --- |
| **RNAi** | **Exp** | **Survival  (% alive  ± SEM)** | **N** | **P value**  **(compared to Control RNAi)** | **Survival  (% alive  ± SEM)** | **N** | **Survival  (% alive  ± SEM)** | **N** | **P value**  **(compared to Control RNAi)** |
| ***tnt-3*** | 1 | 53±8 | 62 | 0.10 (n.s.) | 31±8 | 74 | 81±4 | 64 | 0.001 (**) |
| **(TroponinT)** | 2 | 18±5 | 67 | 0.6 (n.s.) | 14±4 | 57 | 41±4 | 75 | 0.003 (**) |
|  | 3 | 18±5 | 55 | 0.6 (n.s.) | 22±5 | 45 | 49±3 | 47 | 0.004 (**) |
| ***act-1*** | 1 | 35±3 | 56 | 0.7 (n.s.) | 31±8 | 74 | 81±4 | 64 | 0.001 (**) |
| **(Actin)** | 2 | 26±3 | 75 | 0.9 (n.s) | 27±4 | 84 | 65±5 | 71 | 0.001 (**) |
|  | 3 | 39±7 | 78 | 0.8 (n.s.) | 42±6 | 71 | 69±3 | 77 | 0.007 (**) |
| ***act-3*** | 1 | 37±7 | 77 | 0.6 (n.s) | 31±8 | 74 | 81±4 | 64 | 0.001 (**) |
| **(Actin)** | 2 | 29±8 | 79 | 0.8 (n.s) | 27±4 | 84 | 65±5 | 71 | 0.001 (**) |
|  | 3 | 39±6 | 73 | 0.7 (n.s.) | 42±6 | 71 | 69±3 | 77 | 0.007 (**) |

**Table S2.** Survival analysis of wild-type (N2), CF512 (*fer-15(b26); fem-1(hc17))*, or CF2201 (*fer-15(b26*)). Animals were raised on OP50 bacteria until they reached day 1 of adulthood and were then moved to plates containing dsRNA-expressing or control bacteria. As control, bacteria expressing either empty vector or dsRNA against *gfp* were used [empty-vector and *gfp* RNAi clones have equivalent effects on lifespan (data not shown)]. N2, CF512, and CF2201 strains behaved similarly in this assay and are therefore not specified on the table.

On day 3 of adulthood, animals were placed at 36°C for 8 h and survival was scored by gentle prodding with a platinum-wire pick. The experimental repeats for a specific RNAi clone are clustered in adjacent rows and numbered in the first column. The order of the RNAi clone in the table reflects on the number of single experiments in which the RNAi clone induced thermotolerance (compare to **Table S3**, sixth column). We note that the single experimental repeats gave variable survival scores, probably due to fluctuations in the incubator temperature.

Survival is indicated as % alive ± SEM. N*,* number of animals assayed. *P* values (one-way ANOVA or Student’s t-test): n.s., not significant (*P*> 0.05), **P <*0.05, ***P* < 0.01, ****P* < 0.001, n.a., not assayed.

Table S3. Summary of phenotypes observed in animals subjected to RNAi of integrin complex and cytoskeletal components.

| **RNAi** | **Function** | **Appearance**  **(WL RNAi)** | **GFP ::MYO-3 aggregation (WL RNAi)** | **PAT-4 ::GFP integrity  (WL RNAi)** | **Thermotolerance pos/n**  **(AO RNAi)** |
| --- | --- | --- | --- | --- | --- |
| ***pat-4*** | Integrin-linked-kinase (ILK) | Prz | **+** | **+** | **13/13** |
| ***pat-6*** | Parvin, Actopaxin, direct binding partner of PAT-4/ILK | Prz | **+** | **+** | **4/4** |
| ***deb-1*** | Vinculin, requires PAT-4/ILK for proper assembly, attaches actin filaments to integrin complex | - | **-** | **+** | **5/5** |
| ***unc-89*** | Obscurin, homologous to human titin, giant sarcomeric protein | Unc | **+** | **+** | **3/4** |
| ***kin-32*** | Focal adhesion kinase | - | **-** | **-** | **2/3** |
| ***unc-98*** | 37 kDa protein, links integrin-associated proteins to myosin in M-lines | Slightly Unc | **-** | **+** | **2/4** |
| ***unc-95*** | Paxillin | Prz | **+** | **+** | **2/4** |
| ***unc-52*** | Perlecan (ECM) | Prz | **+** | **+** | **1/3** |
| ***myo-3*** | Myosin heavy chain | Unc | **(red.)** | **+** | **1/3** |
| ***unc-97*** | PINCH, direct binding partner of PAT-4/ILK | Prz | **+** | **+** | **1/5** |
| ***unc-112*** | Kindlin, direct binding partner of PAT-4/ILK | Prz, Bmd | **+** | **+** | **0/3** |
| ***ina-1*** | Alpha-integrin | - | **-** | **+** | **0/3** |
| ***pat-2*** | Alpha-integrin | Prz | **+** | **+** | **0/3** |
| ***tnt-3*** | Troponin T | - | **-** | **+** | **0/3** |
| ***act-1*** | Actin | Bmd, Sma | **-** | **-** | **0/3** |
| ***act-3*** | Actin | Bmd, Sma | **-** | **-** | **0/3** |

**Table S3.** Phenotypes were assessed in animals after whole-life (WL) or adult-only (AO) treatment with RNAi clones encoding integrin-signaling complex or cytoskeletal components. Appearance was scored in wild-type animals following WL treatment as follows: Prz, paralyzed; Unc, uncoordinated movement; Bmd, body morphology defects; and Sma, small.

MYO-3::GFP aggregation was examined by fluorescence microscopy in strain RW1596 (*myo-3p::gfp::myo-3*) after WL RNAi treatment. +, many GFP aggregates (compare to **Fig. 1C**); –, no GFP aggregates (compare to **Fig. 1D**); (red.), reduction of GFP intensity.

PAT-4::GFP integrity was determined after WL RNAi treatment by visualizing M-lines and dense bodies in body-wall muscles of strains MAH03 (PAT-4 overexpressor strain: *pat-4p::pat-4::gfp)* and MAH07 (PAT-4 rescue strain: *pat-4; pat-4p::pat-4::gfp)*. +, disorganization or gaps in M-line and dense body structures; –, no clear phenotype scored (data summary of several repeats, see **Fig. S2** for representative images).

Summary of thermotolerance assays performed on wild-type animals subjected to AO RNAi. pos/n, number of repeats in which RNAi induced thermotolerance / total number of thermotolerance experiments (data summary from **Table S2**).

We note that several of the analyzed clones did not induce thermotolerance in any of three trials. Although most of these RNAi clones caused visible phenotypes in assays using WL RNAi (for example, disruption of the PAT-4/ILK expression pattern [**Fig. S2**]), we have not been able to assess whether these RNAi clones can sufficiently reduce protein levels when used in AO RNAi experimental protocols. We have therefore refrained from discussing these clones in further detail in the manuscript.

**Table S4. Survival analysis of transcription factor mutants subjected to *pat-4*/ILK RNAi during adulthood and incubated at 36°**C.

| **Exp** | **Strain** | **RNAi** | **Survival  (% alive  ± SEM)** | **N** | **Control** | **Survival  (% alive  ± SEM)** | **N** | **P value** |
| --- | --- | --- | --- | --- | --- | --- | --- | --- |
| ***daf-16(mu86)* mutants on *pat-4*/ILK RNAi** | | | | | | | | |
| 1 | WT | *pat-4* | 79±7 | 65 | *gfp* | 38±3 | 69 | 0.002 (**) |
|  |  | *daf-2* | 83±6 | 68 | *gfp* | 38±3 | 69 | 0.0005 (***) |
|  | *daf-16* | *pat-4* | 43±7 | 57 | *gfp* | 19±4 | 56 | 0.03 (*) |
|  |  | *daf-2* | 17±4 | 67 | *gfp* | 19±4 | 56 | 0.7 (n.s.) |
| 2 | *daf-16* | *pat-4* | 38±5 | 60 | *gfp* | 67±4 | 63 | 0.004 (**) |
| **Reduction of *daf-16* and *pat-4*/ILK using double RNAi** | | | | | | | | |
| 1 | WT | *pat-4/gfp* | 57±5 | 40 | *gfp* | 23±5 | 52 | 0.003 (**) |
|  |  | *pat-4/daf-16* | 27±7 | 38 | *gfp/daf-16* | 6±2 | 50 | 0.03 (*) |
| 2 | WT | *pat-4/gfp* | 60±9 | 56 | *gfp* | 37±9 | 45 | 0.07 (n.s.) |
|  |  | *pat-4/daf-16* | 60±7 | 63 | *gfp/daf-16* | 28±3 | 54 | 0.006 (**) |
| ***skn-1(zu135)* mutants on *pat-4*/ILK RNAi** | | | | | | | | |
| 1 | WT | *pat-4* | 32±3 | 81 | *gfp* | 7±2 | 77 | 0.0004 (***) |
|  |  | *daf-2* | 52±2 | 61 | *gfp* | 7±2 | 77 | <0.0001 (***) |
|  | *skn-1* | *pat-4* | 27±1 | 55 | *gfp* | 6±4 | 55 | 0.002 (**) |
|  |  | *daf-2* | 39±7 | 67 | *gfp* | 6±4 | 55 | 0.006 (**) |
| 2 | WT | *pat-4* | 33±1 | 57 | *gfp* | 11±2 | 69 | <0.0001 (***) |
|  |  | *daf-2* | 43±5 | 68 | *gfp* | 11±2 | 69 | 0.001 (**) |
|  | *skn-1* | *pat-4* | 37±3 | 45 | *gfp* | 18±5 | 48 | 0.02 (*) |
|  |  | *daf-2* | 37±4 | 67 | *gfp* | 19±4 | 56 | 0.01 (*) |
| **Reduction of *skn-1* and *pat-4*/ILK using double RNAi** | | | | | | | | |
| 1 | WT | *pat-4/gfp* | 70±5 | 76 | *gfp* | 57±1 | 61 | 0.04 (*) |
|  |  | *pat-4/skn-1* | 56±2 | 63 | *gfp/skn-1* | 35±4 | 59 | 0.003 (**) |
| 2 | WT | *pat-4/gfp* | 50±3 | 56 | *gfp* | 27±5 | 72 | 0.004 (**) |
|  |  | *pat-4/skn-1* | 55±6 | 56 | *gfp* | 27±5 | 72 | 0.007 (**) |
| 3 | WT | *pat-4/gfp* | 35±6 | 80 | *gfp* | 16±4 | 83 | 0.04 (*) |
|  |  | *pat-4/skn-1* | 31±5 | 81 | *gfp* | 16±4 | 83 | 0.02 (*) |

Table S4 (continued).

| **Exp** | **Strain** | **RNAi** | **Survival  (% alive  ± SEM)** | **N** | **Control** | **Survival  (% alive  ± SEM)** | **N** | **P value** |
| --- | --- | --- | --- | --- | --- | --- | --- | --- |
|  | ***hsf-1(sy441)* mutants on *pat-4*/ILK RNAi** | | | | | | | |
| 1^1^ | WT | *pat-4* | 59±1 | 59 | *gfp* | 27±3 | 81 | <0.0001 (***) |
|  |  | *daf-2* | 75±3 | 59 | *gfp* | 27±3 | 81 | <0.0001 (***) |
|  | *hsf-1* | *pat-4* | 2±2 | 40 | *gfp* | 6±3 | 117 | 0.3 (n.s.) |
|  |  | *daf-2* | 42±4 | 126 | *gfp* | 6±3 | 117 | 0.0004 (***) |
| 2 | WT after 9h | *pat-4* | 27±2 | 101 | empty vector | 3±1 | 78 | <0.0001 (***) |
|  |  | *pat-6* | 29±4 | 91 | empty vector | 3±1 | 78 | 0.0002 (***) |
|  | *hsf-1* after 7h | *pat-4* | 13±3 | 128 | empty vector | 7±1 | 153 | 0.1 (n.s.) |
|  |  | *pat-6* | 7±2 | 149 | empty vector | 7±1 | 150 | 1.0 (n.s.) |
| 3 | WT  (after 9h) | *pat-4* | 27±2 | 70 | *gfp* | 14±3 | 101 | 0.01 (*) |
|  | *hsf-1* (after 7h) | *pat-4* | 12±3 | 64 | *gfp* | 14±3 | 120 | 0.7 (n.s.) |
|  | *hsf-1* (after 9h) | *pat-4* | 1±1 | 64 | *gfp* | 1±1 | 120 | 1.0 (n.s.) |
| 4 | WT | *pat-4* | 56±13 | 83 | *gfp* | 37±4 | 102 | 0.06 (n.s.) |
|  | *hsf-1* | *pat-4* | 8±2 | 82 | *gfp* | 5±2 | 94 | 0.3 (n.s.) |
| **Reduction of *hsf-1* and *pat-4*/ILK using double RNAi** | | | | | | | | |
| 1 | WT | *pat-4/gfp* | 60±5 | 55 | *gfp* | 42±5 | 53 | 0.03 (*) |
|  |  | *pat-4/hsf-1* | 36±10 | 54 | *gfp/hsf-1* | 43±7 | 47 | 0.6 (n.s.) |
| 2 | WT | *pat-4/gfp* | 45±9 | 42 | *gfp* | 10±1 | 50 | 0.0005 (***) |
|  |  | *pat-4/hsf-1* | 10±3 | 68 | *gfp/hsf-1* | 4±2 | 50 | 0.2 (n.s.) |
| 3 | WT | *pat-4/gfp* | 60±9 | 56 | *gfp* | 37±9 | 45 | 0.07 (n.s.) |
|  |  | *pat-4/hsf-1* | 39±9 | 80 | *gfp/hsf-1* | 26±2 | 72 | 0.2 (n.s.) |

**Table S4.** Survival analysis of N2 wild-type (WT) animals or transcription factor mutants *daf-16(mu86)*, *skn-1(*[*zu135*](http://www.wormbase.org/species/c_elegans/variation/WBVar00275492)*)/*[*nT1*](http://www.wormbase.org/species/c_elegans/rearrangement/nT1), and *hsf-1(sy441)* raised on OP50 bacteria until they reached day 1 of adulthood and then moved to plates expressing dsRNA against *pat-4*/ILK or *gfp*. WT animals were also fed double RNAi clones mixed 1:1 before seeding.

Control bacteria for the double-RNAi experiments contained empty vector, expressed dsRNA for *gfp,* or were a mixture as indicated. Mixed bacteria were analyzed for functionality by scoring phenotypes when animals were subjected to RNAi from hatching: *pat-4* RNAi mixes induced paralysis (Prz), *hsf-1* RNAi induced larval arrest at 25°C, and *skn-1* RNAi yielded mostly dead eggs (data not shown). The functionality of the *daf-16* RNAi clone was not phenotypically assessed. On day 3 of adulthood, animals were incubated at 36°C and survival was scored after 8 h (or the indicated time) by gentle prodding with a platinum-wire pick. The experiment number for each strain is shown in the first column.

Survival is indicated as % alive ± SEM. N*,* number of animals assayed. *P* values (one-way ANOVA or Student’s t-test): n.s., not significant (*P*> 0.05), **P <*0.05, ***P* < 0.01, ****P* < 0.001. Superscript number ^1^ denotes the experiment shown in **Fig. 2G**.

**Table S5. Survival analysis of mutants with defects in neurons involved in thermosensation subjected to *pat-4*/ILK RNAi during adulthood and incubated at 36°C.**

| **Exp** | **Strain** | **RNAi** |  |  | **Control** | | **P value** |
| --- | --- | --- | --- | --- | --- | --- | --- |
|  |  |  | **Survival  (% alive  ± SEM)** | **N** | **Survival  (% alive  ± SEM)** | **N** |  |
| **Adult-only RNAi treatment** | | | | | | | |
| 1^1^ | WT | *pat-4* | 22±1 | 88 | 3±2 | 91 | 0.0001 (***) |
|  |  | *daf-2* | 36±2 | 79 | 3±2 | 91 | <0.0001 (***) |
|  | *gcy-8* | *pat-4* | 6±2 | 82 | 3±2 | 78 | 0.3 (n.s.) |
|  |  | *daf-2* | 21±6 | 79 | 5±2 | 78 | 0.03 (*) |
|  | *ttx-3* | *pat-4* | 9±2 | 94 | 10±3 | 87 | 0.8 (n.s.) |
|  |  | *daf-2* | 32±4 | 86 | 10±3 | 87 | 0.005 (**) |
| 2 | WT | *pat-4* | 23±3 | 39 | 1±1 | 81 | 0.0004 (***) |
|  |  | *daf-2* | 47±4 | 64 | 1±1 | 81 | 0.0006 (***) |
|  | *gcy-8* | *pat-4* | 0±0 | 20 | 4±4 | 33 | 0.4 (n.s.) |
|  |  | *daf-2* | 31±4 | 45 | 4±4 | 33 | 0.003 (**) |
|  | *ttx-3* | *pat-4* | 3±2 | 55 | 2±2 | 66 | 0.7 (n.s.) |
|  |  | *daf-2* | 31±2 | 71 | 2±2 | 66 | <0.0001 (***) |
| 3 | WT | *pat-4* | 22±4 | 94 | 8±2 | 91 | 0.02 (*) |
|  | *gcy-8* | *pat-4* | 9±2 | 85 | 8±2 | 90 | 0.08 (n.s.) |
|  | *ttx-3* | *pat-4* | 1±1 | 69 | 4±1 | 71 | 0.7 (n.s.) |
| **Whole-life RNAi treatment** | | | | | | | |
| 1 | WT | *pat-4* | 47±1 | 111 | 22±5 | 119 | 0.003 (**) |
|  |  | *daf-2* | 52±3 | 113 | 22±5 | 119 | 0.002 (**) |
|  | *gcy-8* | *pat-4* | 12±3 | 109 | 5±2 | 109 | 0.1 (n.s.) |
|  |  | *daf-2* | 51±5 | 134 | 5±2 | 109 | <0.0001 (***) |
|  | *ttx-3* | *pat-4* | 21±3 | 156 | 15±3 | 117 | 0.2 (n.s.) |
|  |  | *daf-2* | 47±2 | 124 | 15±3 | 117 | <0.0001 (***) |
| 2 | WT | *pat-4* | 19±3 | 67 | 4±5 | 67 | 0.04 (*) |
|  |  | *daf-2* | 50±4 | 76 | 4±5 | 67 | 0.0004 (***) |
|  | *gcy-8* | *pat-4* | 6±4 | 70 | 5±3 | 67 | 0.9 (n.s.) |
|  |  | *daf-2* | 16±1 | 70 | 5±3 | 67 | 0.01 (*) |
|  | *ttx-3* | *pat-4* | 5±2 | 75 | 4±2 | 69 | 0.7 (n.s.) |
|  |  | *daf-2* | 26±4 | 61 | 4±2 | 69 | 0.0002 (***) |

**Table S5 (continued).**

| **Exp** | **Strain** | **RNAi** |  | |  | **Control** | | **P value** |
| --- | --- | --- | --- | --- | --- | --- | --- | --- |
|  |  |  | **Survival  (% alive  ± SEM)** | | **N** | **Survival  (% alive  ± SEM)** | **N** |  |
| 3 | WT | *pat-4* | 35±4 | 61 | | 15±5 | 54 | 0.02 (*) |
|  | *gcy-8* | *pat-4* | 11±2 | 66 | | 9±4 | 76 | 0.7 (n.s.) |
|  | *ttx-3* | *pat-4* | 24±3 | 92 | | 22±1 | 74 | 0.6 (n.s.) |
| 4 | WT | *pat-4* | 48±7 | 63 | | 20±6 | 80 | 0.02 (*) |
|  | *gcy-8* | *pat-4* | 6±3 | 75 | | 8±1 | 66 | 0.6 (n.s.) |
|  | *ttx-3* | *pat-4* | 14±3 | 55 | | 28±6 | 67 | 0.08 (n.s.) |
| 5 | WT | *pat-4* | 23±3 | 75 | | 11±4 | 82 | 0.05 (*) |
|  | *gcy-8* | *pat-4* | 19±8 | 54 | | 15±3 | 60 | 0.7 (n.s.) |
|  | *ttx-3* | *pat-4* | 4±3 | 71 | | 13±5 | 72 | 0.2 (n.s.) |

**Table S5.** Survival analysis of N2 wild-type (WT) animals or *gcy-8(oy44)* and *ttx-3(ks5)* mutants (defects in neurons involved in thermosensation). Animals were fed bacteria expressing dsRNA against *pat-4*/ILK, *daf-2*/InR, or *gfp* (control).

For adult-only RNAi experiments (upper section), the animals were raised on OP50 bacteria until they reached day 1 of adulthood and were then moved to plates containing dsRNA-expressing bacteria. For whole-life RNAi experiments (lower section), animals were fed dsRNA-expressing bacteria from hatching. Animals were incubated at 36°C for 8 h and survival was scored by gentle prodding with a platinum-wire pick. The experiment number for each strain is shown in the first column.

Survival is indicated as % alive with SEM. N*,* observed deaths. *P* values (one-way ANOVA or Student’s t-test): n.s., not significant; **P <*0.05, ***P* < 0.01, ****P* < 0.001. Superscript number ^1^ denotes the experiment shown in **Fig. 4B**.

**Table S6. Survival analysis of *gon-2* mutants subjected to *pat-4*/ILK RNAi during adulthood and incubated at 36°C.**

| **Exp** | **Strain** | **RNAi** | **Survival  (% alive  ± SEM)** | **N** | **Control RNAi**  91 | | **P value** | **P value of strains on Control RNAi** |
| --- | --- | --- | --- | --- | --- | --- | --- | --- |
|  |  |  |  |  | **Survival  (% alive  ± SEM)** | **N** |  |  |
| **Thermorecovery of *gon-2* mutants subjected to adult-only RNAi treatment** | | | | | | | | |
| 1 | WT | *pat-4* | 63±3 | 81 | 42±2 | 91 | 0.006 (**) | 0.2 (n.s.) |
|  | *gon-2* | *pat-4* | 30±5 | 57 | 30±7 | 67 | 1.0 (n.s.) |  |
| 2 | WT | *pat-4* | 48±1 | 109 | 28±2 | 106 | 0.0001 (***) | 0.2 (n.s) |
|  | *gon-2* | *pat-4* | 34±6 | 72 | 22±3 | 68 | 0.1 (n.s.) |  |
| 3 | WT | *pat-4* | 68±4 | 53 | 42±4 | 58 | 0.002 (**) | 0.002 (**) |
|  | *gon-2* | *pat-4* | 68±46 | 51 | 72±5 | 57 | 0.6 (n.s.) |  |
| **Thermotolerance of *gon-2* mutants subjected to adult-only RNAi treatment** | | | | | | | | |
| 1 | WT | *daf-2* | 47±4 | 68 | 15±1 | 70 | 0.0002 (***) | 0.01 (*) |
|  |  | *pat-4* | 38±2 | 86 | 15±1 | 70 | <0.0001 (***) |  |
|  | *gon-2* | *daf-2* | 59±6 | 93 | 7±2 | 47 | 0.06 (n.s.) |  |
|  |  | *pat-4* | 22±6 | 42 | 7±2 | 47 | 0.04 (*) |  |
| 2 | WT | *pat-4* | 43±3 | 72 | 14±3 | 71 | 0.0005 (***) | 0.5 (n.s.) |
|  | *gon-2* | *pat-4* | 16±3 | 72 | 17±3 | 82 | 0.8 (n.s) |  |
| **Thermorecovery of *gon-2* mutants subjected to whole-life RNAi treatment** | | | | | | | | |
| 1 | WT | *pat-4* | 76±3 | 70 | 48±5 | 65 | 0.003 (**) | 0.2 (n.s.) |
|  | *gon-2* | *pat-4* | 78±6 | 78 | 62±7 | 60 | 0.2 (n.s) |  |
| 2 | WT | *pat-4* | 40±4 | 139 | 8±2 | 109 | 0.0004 (***) | 0.2 (n.s) |
|  | *gon-2* | *pat-4* | 13±3 | 97 | 14±3 | 104 | 0.8 (n.s.) |  |
| 3 | WT | *pat-4* | 72±9 | 26 | 10±6 | 33 | 0.001 (**) | 0.1 (n.s.) |
|  | *gon-2* | *pat-4* | 66±6 | 27 | 39±14 | 36 | 0.1 (n.s.) |  |
| **Thermotolerance of *gon-2* mutants subjected to whole-life RNAi treatment** | | | | | | | | |
| 1 | WT | *daf-2* | 55±5 | 68 | 17±1 | 67 | 0.0003 (***) | 0.003 (**) |
|  |  | *pat-4* | 55±3 | 78 | 17±1 | 67 | <0.0001 (***) |  |
|  | *gon-2* | *daf-2* | 42±3 | 98 | 28±2 | 61 | 0.008 (**) |  |
|  |  | *pat-4* | 25±2 | 54 | 28±2 | 61 | 0.3 (n.s.) |  |
| 2 | WT | *pat-4* | 60±4 | 41 | 21±8 | 43 | 0.005 (**) | 0.2 (n.s.) |
|  | *gon-2* | *pat-4* | 33±12 | 62 | 40±10 | 30 | 0.7 (n.s) |  |

**Table S6 (continued).**

| **Exp** | **Strain** | **RNAi** | **Survival  (% alive  ± SEM)** | **N** | **Control RNAi**  91 | | **P value** | **P value of strains on Control RNAi** |
| --- | --- | --- | --- | --- | --- | --- | --- | --- |
|  |  |  |  |  | **Survival  (% alive  ± SEM)** | **N** |  |  |
| **Thermotolerance of *gon-2* mutants subjected to whole-life RNAi treatment** | | | | | | | | |
| 3 | WT | *pat-4* | 50±2 | 71 | 27±3 | 76 | 0.0007 (***) | 0.4 (n.s.) |
|  | *gon-2* | *pat-4* | 22±3 | 109 | 32±5 | 81 | 0.1 (n.s) |  |

**Table S6.** Survival analysis of N2 wild-type (WT) and *gon-2(q388)* animals fed bacteria expressing dsRNA against *pat-4*/ILK, *daf-2*/InR, or *gfp* (control). The temperature-sensitive *gon-2* mutant develops without gonad structures when raised at the restrictive temperature of 25°C during development .

For adult-only RNAi experiments, animals were raised on OP50 bacteria at 25°C until they reached day 1 of adulthood and then moved to 20°C on plates containing dsRNA-expressing bacteria. Survival of animals on day 3 of adulthood was scored in two ways. For thermotolerance experiments, animals were incubated at 36°C for 8 h and survival was scored by gentle prodding with a platinum-wire pick. For thermorecovery experiments, animals were incubated at 36°C for 8 h, then placed at 20°C for 20 h of recovery, and survival was scored by voluntary movement of animals on plates (see Methods). For whole-life RNAi experiments, animals were fed dsRNA-expressing bacteria from hatching at 25°C, and the experiments were performed on day 1 of adulthood in a similar manner. Downregulation of *pat-4*/ILK mRNA levels upon RNAi treatment was confirmed using Q-PCR in *gon-2* mutants (data not shown).
The experiment number for each strain is shown in the first column.

Survival is indicated as % alive with SEM. N, number of animals assayed. *P* values (Student’s t-test): n.s., not significant (*P*> 0.05), **P <*0.05, ***P* < 0.01, ****P* < 0.001.

**Table S7. *C. elegans* strains used in this study.**

| **Name** | **Genotype** | **Strain origin** |
| --- | --- | --- |
| **Published strains used in this study** | | |
| N2 | Wild-type (WT) | Hansen lab, originated from Kenyon lab |
| AM140 | *rmIs132[unc-54p::Q35::YFP] I* | CGC^1^ |
| BC13546 | *dpy-5(e907) I/?; sEx13546[pat-4p::gfp]* | Baillie lab |
| CF512 | *fer-15(b26) II; fem-1(hc17) IV* | Kenyon lab |
| CF1037 | *daf-16(mu86) I* | Kenyon lab |
| CF1553 | *muIs84[sod-3p::gfp]* | Kenyon lab |
| CF1874 | *daf-16(mu86) I; muIs84[sod-3p::gfp]* | Kenyon lab |
| CF2201 | *fer-15(b26) II* | Kenyon lab |
| CF2253 | *gon-2(q388) I* | Kenyon lab |
| CF2495 | *hsf-1(sy441) I* | Kenyon lab |
| EU31 | *skn-1(zu135) IV / nT1[unc-?(n754) let-?] (IV, V)* | CGC |
| FK134 | *ttx-3(ks5) X* | CGC |
| HC196 | *sid-1(qt9) V* | Hunter lab |
| IK800 | *gcy-8(oy44) IV* | CGC |
| MAH23 | *rrf-1(pk1417) I* | Hansen lab |
| OH1098 | *otIs133[ttx-3p::RFP +unc-4(+)] II* | CGC |
| RW1596 | *myo-3(st386) V; stEx30[myo-3p::gfp::myo-3 + rol-6(su1006)]* | CGC |
| TJ001 | *[gst-4p::gfp]* | Blackwell lab |
| TJ375 | *gpIs1[hsp-16.2p::gfp]* | CGC |
| TV2070: | *wyEx795[unc122p::GFP; gcy-8p::mCh; ttx-3p::CFP]* | Shen lab |
| WB125 | *pat-4(st551) III; zpEx194[pat-4p::pat-4::gfp + rol-6(su1006)]* | Williams lab |
| **New strains created for this study** | | |
| AGD638 | *sid-1(qt9) V; uthIs206[rab-3p::tomato::unc-54-3’UTR; rab-3p::sid-1::unc-54-3’UTR]* | |
| AGD855 | *sid-1(qt9) V; uthIs237[myo-3p::tomato::unc-54-3’UTR; myo-3p::sid-1::unc-54-3’UTR]* | |
| MAH03 | *zpEx194[pat-4p::pat-4::gfp + rol-6(su1006)]* | |
| MAH07: | *pat-4(st551) III; zpEx194[pat-4p::pat-4::gfp + rol-6(su1006)]* | |
| MAH265 | *hsf-1(sy441) I;* *muIs84[sod-3p::gfp]* | |
| MAH268 | *dpy-5(e907) I/?; otIs133[ttx-3p::rfp +unc-4(+)] II; sEx13546[pat-4p::gfp]* | |
| MAH269 | *hsf-1(sy441) I;* *gpIs1[hsp-16.2p::gfp]* | |
| MAH274 | *dpy-5(e907) I/?; sEx13546[pat-4p::gfp]; wyEx795[unc122p::gfp; gcy-8p::mCh;  ttx-3p::cfp]* | |
| MAH295 | *ttx-3(ks5) X; gpIs1[hsp-16.2p::gfp]* | |
| MAH296 | *gcy-8(oy44) IV; gpIs1[hsp-16.2p::gfp]* | |

^1^: *Caenorhabditis* Genetics Center (CGC)

Table S8. Sequences of quantitative RT-PCR primers used in this study.

| **Gene** | **Primer sequence 5’ 🡪 3’** | |
| --- | --- | --- |
| *pat-4* | Fwd | CCT ATT CGC CTG CCT GGA T |
|  | Rev | CAT ATT ACG GGC GAT TCC AG |
| *hsf-1* | Fwd | CGA AAG ATG ACT CCA CTG TCC |
|  | Rev | GTC CTC CAC AGT TCT TGC C |
| *unc-23* | Fwd | GCC AGT CGT GAA CAT ACC AAT CAA AAT ACT TGG TC |
|  | Rev | GGC AGG TGG CTC CAG CAG TTT CTT CGT ACG AAC AG |
| *aip-1* | Fwd | GGC GGA GTT CCC AAA TCT CGG AAA GCA CTG TG |
|  | Rev | GGT GCA GTT GGA ATT GGA ATT TCT TGT TTG ATG C |
| *sod-3* | Fwd | GCT GCA ATC TAC TGC TCG CAC TGC TTC AAA GC |
|  | Rev | GGC AAA TCT CTC GCT GAT ATT CTT CCA GTT GGC |
| *hsp-16.2* | Fwd | ACT TTA CCA CTA TTT CCG TCC AGC |
|  | Rev | CCT TGA ACC GCT TCT TTC TTT G |
| *sip-1* | Fwd | ATG TCT TCT CTC TGC CCA TAC ACT GGC CG |
|  | Rev | TTA GTG CTT TCC GGT GGT GGT GGT GCT GG |
| *hsp-70 (1) (C12C8.1)* | Fwd | ACT CAT GTG TCG GTA TTT ATC |
|  | Rev | ACG GGC TTT CCT TGT TTT |
| *hsp-70 (2) (F44E5.4)* | Fwd | AAT GAA CCA ACT GCT GCT GCT CTT |
|  | Rev | TGT CCT TTC CGG TCT TCC TTT TG |
| *hsp-16.49* | Fwd | GCT CAT GCT CCG TTC TCC ATA TTC TGA TTC AAA TGC |
|  | Rev | GCA ACA AAA TTG ATC GGA ATA GAA CGT GAT GAG |
| *hsp-16.1* | Fwd | GTC ACT TTA CCA CTA TTT CCG TCC AGC TCA ACG TTC |
|  | Rev | CAA CGG GCG CTT GCT GAA TTG GAA TAG ATC TTC C |
| *hsp-12.6* | Fwd | ATG ATG AGC GTT CCA GTG ATG GCT GAC G |
|  | Rev | TTA ATG CAT TTT TCT TGC TTC AAT GTG AAG AAT TCC |
| *cdr-1* | Fwd | TCT TCT CTC AAT TGG CAA CTG |
|  | Rev | TTT GGG TAA ACT TCA TGA CGA |
| *daf-21* | Fwd | CGC TAC CAG GCA CTC ACC GAG |
|  | Rev | GGA CAA GCT CTT GTA GAA CTC AG |
| **Housekeeping genes** | | |
| *ama-1* | Fwd | TGG AAC TCT GGA GTC ACA CC |
|  | Rev | CAT CCT CCT TCA TTG AAC GG |
| *cdc-42* | Fwd | CTG CTG GAC AGG AAG ATT ACG |
|  | Rev | CTC GGA CAT TCT CGA ATG AAG |
| *nhr-23* | Fwd | CAG AAA CAC TGA AGA ACG CG |
|  | Rev | CGA TCT GCA GTG AAT AGC TC |
| *pmp-3* | Fwd | GTT CCC GTG TTC ATC ACT CAT |
|  | Rev | ACA CCG TCG AGA AGC TGT AGA |

**SUPPORTING FIGURE LEGENDS**

**Figure S1. *pat-4*/ILK mRNA levels are reduced in *C. elegans* by whole-life and adult-only RNAi treatments.**

(**A**) Quantitative RT-PCR was performed on total RNA from 2-day-old N2 wild-type animals raised from hatching on *pat-4*/ILK dsRNA-expressing or control bacteria (whole-life RNAi). mRNA expression levels were normalized to the housekeeping genes *ama-1* and *nhr-23*.

(**B**) Quantitative RT-PCR was performed on total RNA from 3-day-old N2 wild-type animals fed *pat-4*/ILK dsRNA-expressing or control bacteria from day 1 on (adult-only RNAi). mRNA expression levels were normalized to the housekeeping genes *ama-1,* *nhr-23, cdc-42 and pmp-3*. The results from both A and B are the Mean + SEM of three independent experiments. **P* < 0.05, ****P* < 0.001 (Student’s *t-*test)**.** All experiments were performed at 20°C.

**Figure S2. Effects of reduction of integrin complex components in *C. elegans* expressing GFP-tagged PAT-4/ILK.**

Animals expressing PAT-4::GFP under the control of the endogenous *pat-4* promoter were raised from hatching on bacteria containing empty vector or expressing dsRNA targeting integrin-signaling complex and cytoskeletal proteins. Animals were imaged on day 1 of adulthood. Scale bar = 5 μm. The experiment was repeated in wild-type animals and *pat-4(st551)* mutants with similar results. The α-integrin (*ina-1)* and troponin T (*tnt-3)* RNAi disrupted the PAT-4::GFP structure to variable extents, with the most severe phenotypes shown here.

**Figure S3. Effects on stress-inducible GFP reporters following inhibition of *pat-4*/ILK in adult *C. elegans* transcription factor mutants .**

(**A**) Fluorescence micrographs of wild-type animals expressing *sod-3p::gfp* (top panel), *gst-4p::gfp* (middle panel), or *hsp-16.2p::gfp* (bottom panel). Animals were transferred to *pat-4*/ILK dsRNA-expressing or control bacteria on day 1 and images were captured on day 3.

(**B**) Fluorescence micrographs of *daf-16(mu86)* mutants expressing *sod-3p::gfp* (top panel), wild-type animals expressing *gst-4p::gfp* and subjected to adult-only *skn-1*/Nrf dsRNA (double-RNAi approach; middle panel), or *hsf-1(sy441)* mutants expressing *hsp-16.2p::gfp* (bottom panel). Animals were transferred to *pat-4*/ILK dsRNA-expressing or control bacteria on day 1 and images were captured on day 3.

(**C**) Fluorescence micrographs of *hsf-1(sy441)* mutants expressing *sod-3p::gfp* (top panel) and wild-type animals expressing *gst-4p::gfp* and *hsf-1* dsRNA (double-RNAi approach; middle panel). Animals were transferred to *pat-4*/ILK dsRNA-expressing or control bacteria on day 1 and images were captured on day 3. A consistent increase was observed in *gst-4p::gfp* expression in animals subjected to *hsf-1* RNAi by day 3 of adulthood; however, this increase was not observed upon whole-life treatment on day 1 (data not shown). The exposure time was 1 s for *sod-3p::gfp* and *hsp-16.2p::gfp* and 600 ms for *gst-4p::gfp*. Scale bars = 200 μm. Prior to imaging of the *hsp-16.2p::gfp* reporter, animals were incubated for 1 h at 36°C followed by 2 h at 20°C.

(**D**) Quantification of the GFP intensity of the animals shown in (**A**)–(**C**). Results are the Mean + SEM of N = 9-12*. *P* < 0.05, ****P* < 0.001 (two-way ANOVA). The experiments were repeated at least three times with similar results.

**Figure S4. *pat-4*/ILK mRNA levels are reduced in *hsf-1* mutants**

Quantitative RT-PCR was performed on total RNA from 2-day-old *hsf-1(sy441)* mutants raised from hatching on *pat-4*/ILK dsRNA-expressing or control bacteria. mRNA expression levels were normalized to the housekeeping genes *ama-1,* *nhr-23 and pmp-3*. *pat-4*/ILK RNAi caused complete paralysis of these animals. The results are the Mean + SEM of three independent experiments. ****P* < 0.001 (Student’s *t*-test). All experiments were performed at 20°C.

**Figure S5. Reduction of *pat-4*/ILK in *gcy-8* and *ttx-3* mutants does not increase transcription of HSF-1 target genes.**

Quantitative RT-PCR was performed on total RNA from 2-day-old *gcy-8(oy44)* (**A**) and *ttx-3(ks5)* (**B**) mutants raised from hatching on *pat-4*/ILK dsRNA-expressing or control bacteria. mRNA expression levels were normalized to the housekeeping genes *ama-1* and *nhr-23*. *pat-4*/ILK RNAi caused complete paralysis of these animals.

(**C**) Quantitative RT-PCR was performed on total RNA from 3-day-old tissue-specific RNAi strains (neurons: *sid-1(qt9); rab-3p::sid-1*, muscle: *sid-1(qt9); myo-3p::sid-1*, see **Table S7** for full genotype) cultured on *pat-4*/ILK dsRNA-expressing or control bacteria from day 1 of adulthood. mRNA expression levels were normalized to the housekeeping genes *ama-1, nhr-23, cdc-42* and *pmp-3*. We note that we have analyzed these strains on a panel of RNAi clones targeting genes expressed in various tissues (*unc-112* (body-wall muscle), *elt-2* (intestine), *bli-3* (hypodermis), *gld-1* (germline) ) to test their specificity of RNAi processing. The muscle-specific RNAi strain only displayed phenotypes upon body-wall-muscle targeting RNAi treatment (*i.e.*, *unc-112* and *pat-4* RNAi). The neuronal-specific RNAi strain showed some effects when fed the *elt-2* RNAi clone, suggesting some intestinal RNAi-processing capabilities. However, since *pat-4*/ILK is not expressed in the intestine, this should not affect our conclusions drawn from the experiments using this strain.

All results are the mean + SEM of three independent experiments. All results were statistically not significant unless indicated as **P* < 0.05, ***P* < 0.01, ****P* < 0.001 (one-way ANOVA). All experiments were performed at 20°C.

**Figure S6. Thermotactic behavior is intact in *C. elegans* with reduced PAT-4/ILK levels.**

Thermotactic behavior of N2 wild-type (WT) animals (**A**), *gcy-8* mutants (**B**), and *ttx-3* mutants (**C**) was assayed in agar chunks at temperatures between 20°C and 36°C or between 4°C and 20°C. Animals were fed *pat-4*/ILK dsRNA-expressing or control bacteria from day 1 of adulthood, and the assay was performed on day 3. The results are displayed as % of total animals per plate ± SEM with n = 3 and population of N = 12-20 per plate. The experiment was repeated three times with similar results.

**Figure S7. Whole-life inhibition of *pat-4*/ILK increases aggregation of polyglutamine-expansion repeats in *C. elegans*.**

(**A**) Fluorescence micrographs of *C. elegans* expressing *unc-54p::Q35::yfp* in body-wall muscles. Animals were raised from hatching on *pat-4*/ILK dsRNA-expressing or control bacteria and images were captured on day 1 of adulthood.

(**B**) Quantification of YFP aggregates per animal. Results are the Mean + SEM of N = 10-12*.* ****P* < 0.001 (Student’s *t*-test). The experiments were repeated at least three times with similar results.
